# Imaging based body composition profiling and outcomes after oncologic liver surgery

**DOI:** 10.3389/fonc.2022.1007771

**Published:** 2022-12-08

**Authors:** Lorenzo Bernardi, Raffaello Roesel, Filippo Vagelli, Pietro Majno-Hurst, Alessandra Cristaudi

**Affiliations:** ^1^ Department of Visceral Surgery, Lugano Regional Hospital, Ente Ospedaliero Cantonale (EOC), Lugano, Switzerland; ^2^ University of Pisa, Medical School, Pisa, Italy; ^3^ University of Southern Switzerland (USI), Department of Biomedical Science, Lugano, Switzerland

**Keywords:** cancer imaging, muscle, sarcopenia, body composition, liver surgery, liver resection

## Abstract

Body composition profiling is gaining attention as a pre-operative factor that can play a role in predicting the short- and long- term outcomes of patients undergoing oncologic liver surgery. Existing evidence is mostly limited to retrospective and single-institution series and in many of these studies, the evaluation of body composition is based on parameters which are derived from CT-scan imaging. Among body composition phenotypes, sarcopenia is the most well studied but this is only one of the possible profiles which can impact the outcomes of oncologic hepatic surgery. Interest has recently grown in studying the effect of sarcopenic obesity, central obesity, or visceral fat amount, myosteatosis, and bone mineral density on -such patients. The objective of this review is to summarize the current evidence on whether imaging-based parameters of body composition have an impact on the outcome of patients undergoing liver surgery for each of the most frequent indications for liver resection in clinical practice: hepatocellular carcinoma (HCC), cholangiocarcinoma (CCA), and colorectal liver metastases (CRLM).

## Introduction

Body composition profiling is gaining attention as a pre-operative factor which can play a role in predicting the short- and long- term outcomes of patients undergoing oncologic liver surgery.

The current evidence is mostly limited to retrospective trials and single-institution series. In many of these studies, the evaluation of body composition profiling is based on parameters obtained from pre-operative CT-scan imaging, mainly focusing on the presence of sarcopenia, a condition of both low muscle mass and low muscle function (strength or performance) (1, 2).

More in detail, three meta-analysis and 3 reviews published in the period 2015 – 2022 used sarcopenia (defined as the loss of skeletal muscle quantity or quality) as the body composition profile of interest. The three meta-analysis studied the impact of sarcopenia on the outcomes of liver surgery for primary liver cancers such as hepatocellular carcinoma (HCC) and cholangiocarcinoma (CCA) suggesting that at least in the case of HCC, sarcopenia negatively effects the post-operative outcomes and possibly have a detrimental effect on long-term survival (3–5). In a review from 2015 pre-operative sarcopenia was indicated as an independent negative prognostic factor for short- and long-term outcomes after hepatectomy in patients with liver cancers; the study was limited by the small number of studies included and by a general lack of data (6). Two others more recent reviews pointed out the detrimental effect of sarcopenia on patients with HCC undergoing hepatectomy and treatments in general (7, 8).

While for HCCs the literature seems to be therefore more consistent, indicating a negative prognostic role of sarcopenia independently of cancer stage, liver function, or allocated treatment, the relation between body composition and the outcomes of surgery for others indications remains poorly investigated (9, 10). Moreover, sarcopenia is only one of the possible imagine-based body composition parameters which can impact the outcomes of oncologic liver surgery. Recently, interest has grown in studying the effect of sarcopenic obesity, central obesity, or visceral fat amount, myosteatosis, and bone mineral density on such patients (11–13).

The aim of this study is to review and summarize the current evidence available on the impact of imaging-based body composition on patients undergoing liver surgery for primary or secondary malignant liver tumors in term of surgical and oncologic outcome. Existing evidence will be synthetized for each of the most frequent indications for liver resection in clinical practice: hepatocellular carcinoma (HCC), cholangiocarcinoma (CCA), and colorectal liver metastases (CRLM).

## Methods

This updated narrative review was performed in accordance with the guidelines of preferred reporting items for systematic reviews and metanalysis (PRISMA) 2020.

### Literature search strategy and study identification

A systematic literature search of PubMed (MEDLINE) and Cochrane Library databases was performed independently by three researchers (LB, RR, FV). The following combination of MESH terms was used: “sarcopenia, body composition, liver surgery”. Research was restricted for papers published from January 2010. Moreover, relevant articles as well as references from reviews and meta-analysis published in the period of interest were manually screened and added according to their relevance to the topic.

### Eligibility criteria and selection process

The population, intervention, comparator, outcome, timing and setting (PICOTS) strategy was used to formulate study questions and to select studies according to the following criteria.

### Population/intervention

Studies were considered eligible for inclusion if including adult patients undergoing liver resection (LR) for any indication (benign/malignant), excluding living donors for living donor liver transplantation (LDLT). Studies were excluded when the body composition was not evaluated by imaging-based parameters (i.e., BMI only, metabolic syndrome, etc.).

### Comparator

As comparator we included any surrogate of body composition, or any marker or imaging parameter used to evaluate body composition of patients undergoing liver surgery.

### Outcome/timing

The endpoints were short-term outcomes specifically intended as post-operative morbidity, major morbidity, specific liver-related morbidity (defined as composite endpoints or according to author’s definitions), post-hepatectomy liver failure (defined with ISGLS criteria or by other systems of classification when specified), mortality (within 30- or 90-days after surgery). Long-term outcomes were overall survival (OS), recurrence-free or disease-free survival (RFS/DFS) (14).

### Setting

We considered for inclusion any comparative study design (case–control and cohort studies, prospective or retrospective) except study protocols, narrative or systematic reviews, common overviews, letters, case reports, experimental (animal model) studies, and conference abstracts, case series of fewer than 10 patients.

The reference list of articles in full text was screened to find relevant articles. No language restrictions were applied. Studies not meeting the inclusion criteria or not reporting the outcomes of interest were excluded as well as studies not including patients underwent liver surgery, studies including pediatric populations and duplicated articles. Non-comparative articles were also excluded.

1)By PICOTS strategy, 3 study questions were elaborated:
Does body composition effect short terms outcomes after liver surgery? (Post-operative morbidity, Major-morbidity, liver-specific morbidity, mortality)
• Liver resection irrespective of the indication
• Hepatocellular carcinoma (HCC),
• Cholangiocarcinoma (CCA),
• Colorectal liver metastases (CRLM)
2) Does body composition affect survival after liver resection?
• Hepatocellular carcinoma (HCC),
• Cholangiocarcinoma (CCA),
• Colorectal liver metastases (CRLM)
3) Does body composition have any impact on the administration of neoadjuvant or adjuvant treatment in CRLM patients?

### Data collection and outcome measure

Three reviewers screened article titles and abstracts according to the inclusion and exclusion criteria, and the resulting full-text articles were further assessed for eligibility based on the inclusion criteria by the same reviewers. Disagreements between investigators were resolved by internal discussion. For each record included, baselined data of the populations of interest were collected, as well as data on the intra- and post-operative details.

### Statistical analysis

Descriptive statistics were performed, and data expressed as mean (SD) or median (range) where appropriate. Statistical significance was set at p<0.05. In the case of missing data, mean and standard deviation were estimated from available values.

## Results

**Figure 1:**
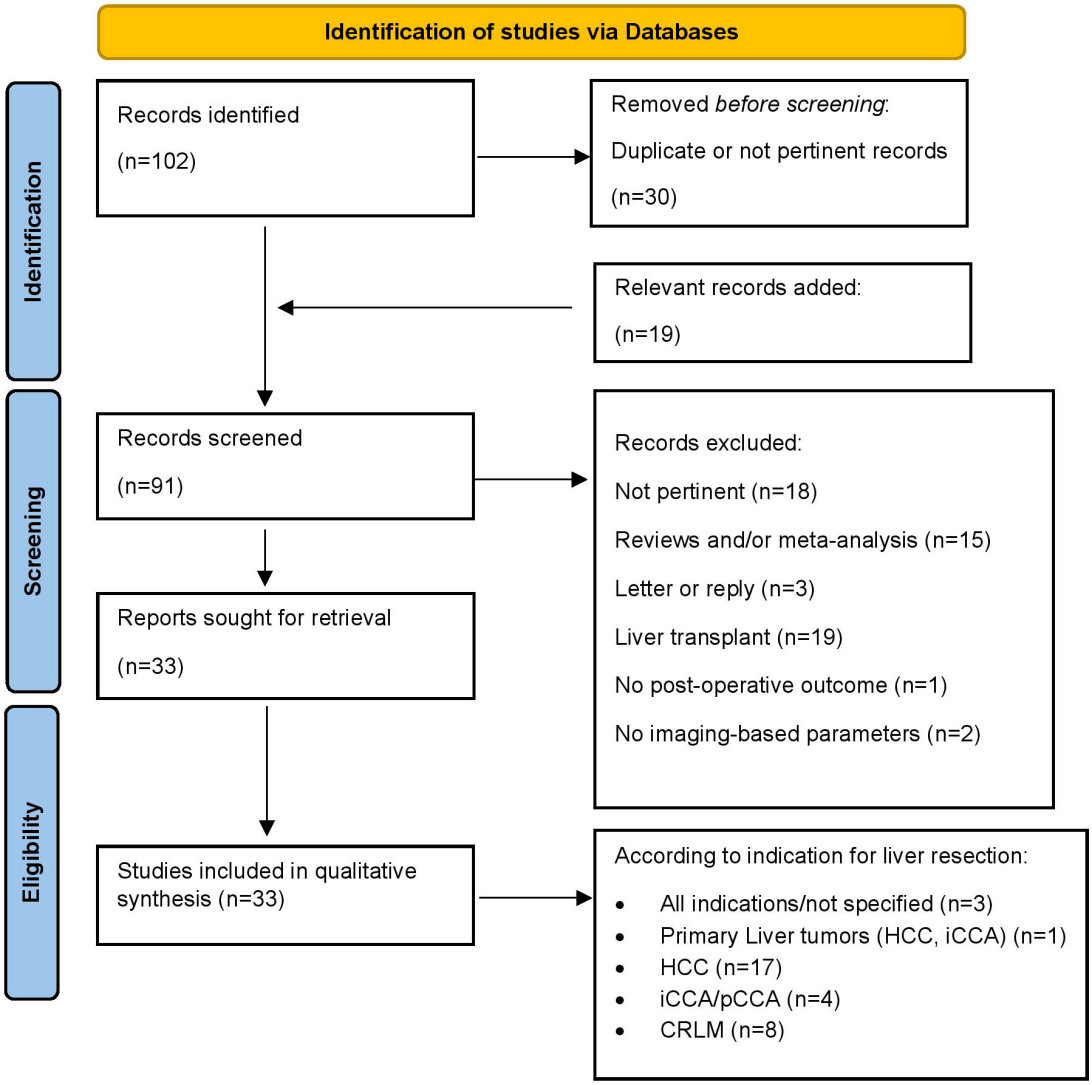
Identification of studies via Databases.

The study flow is summarized in **Figure 1**. One-hundred-two records were selected by mesh-words search and other 19 articles were selected following manual review of the literature. After proper screening and selection, 33 studies fulfilled the eligibility criteria and were included in the qualitative analysis. To easier reader’s comprehension, a synopsis of the body composition patterns which are used in the literature included, of their definition and methods of measure is displayed in **Table 1**. The studies included in the qualitative analysis and their characteristics are summarized in **Table 2**.

**Table 1:** Synopsis of body composition patterns, definitions and measures used in the literature.

Body composition patterns	*Definition*	Methods used to measure body composition		Method-related definition
Sarcopenia (1,2)	*Low skeletal muscle quantity or quality in the presence of low muscle strength*	Skeletal muscle index (SMI) or skeletal muscle mass (SMM)	The cross-sectional areas (cm2) of skeletal muscles on an axial section through L3 vertebrae, is measured by manual outlining on preoperative CT scans with a preestablished density threshold (i.e., −29 to +150 HU) and then normalized for height to obtain the SMI or SMM (cm2/m2).	Low SMI or SMM
		Psoas muscle index (PMI)	The cross-sectional areas of the bilateral psoas muscles measured by manual tracing in preoperative CT images at L3 vertebrae is normalized for height to obtain the PMI (cm2/m2).	Low PMI
		Skeletal muscle area (SMA)	The cross-sectional area of the skeletal muscle is measured by manual tracing in preoperative CT images at L3 vertebrae and normalized for height to obtain the normalized SMA (mm2/m2).	Low SMA
		Total psoas area (TPA)	The cross-sectional area of the bilateral psoas muscles is measured by manual tracing in preoperative CT images at L3 vertebrae and normalized for height to obtain the normalized TPA (mm2/m2).	Low TPA
		Total psoas volume (TPV)	TPV (cm3) is measured by hand tracing the borders of the entire psoas muscles for the total psoas length in preoperative CT scan and then normalizing by height (cm3/m)	Low TPV
		Intramuscular adipose tissue content (IMAC)	The cross-sectional area of CT scan at L3 vertebrae is measured to identify abdominal adipose tissue based on specific attenuation (Visceral adipose tissue: -150 to -50 HU). IMAC = CT attenuation of the multifidus and/or erector spinae muscles (HU)/CT attenuation of subcutaneous adipose tissue (HU).	High IMAC
		Muscle attenuation (MA)	The mean CT attenuation value of the cross-sectional area of the skeletal muscle (HU) in preoperative CT scan image at L3 vertebrae.	Low MA
Sarcopenic obesity	Low skeletal muscle quantity or quality and obesity	Skeletal muscle index (SMI) (cm2/m2) and body mass index (BMI) or body fat percentage (BFP).	SMI is measured as abovementioned. Obesity is measured by BMI or by BFP. Body fat percentage = [total body weight (kg) – fat-free body mass(kg)]/total body weight(kg)x100%. Total fat-free body mass (kg) = 0.30x(skeletal muscle at L3 [cm2] + 6.06).	Low SMI + obesity
Central or visceral obesity or visceral adiposity or visceral fat amount (11)	Visceral adipose tissue content	Visceral (intra-abdominal) adipose tissue index (VATI)	The cross-sectional area (cm2) of adipose tissue is measured by manual outlining in preoperative CT scans at L3 vertebrae or at the level of the umbilicus based on specific attenuation (-150 to -50 HU) and normalized for height (m) to obtain the VATI (cm2/m2).	high VATI
		Visceral fat area (VFA)	The cross-sectional area (cm2) of visceral adipose tissue is measured in preoperative CT scans at L3 vertebrae or at the level of the umbilicus based on specific attenuations (-190 to -30 HU) and normalized for height (m) to obtain VFA (cm2/m2).	High VFA
		visceral to subcutaneous fat area ratio (VSR)	The cross-sectional area (cm2) of subcutaneous and visceral adipose tissue are measured in CT scans at L3 vertebrae or at the level of the umbilicus based on specific attenuations (-190 to -30 HU for subcutaneous adipose tissue and -150 to -50 HU for visceral adipose tissue), then normalized for height (m). VSR= VAT area (cm2)/SAT area (cm2).	High VSR
Myosteatosis (12)	Intramuscular fat deposits	Psoas muscle attenuation (PMA) or Skeletal muscle radiation attenuation (SM-RA)	The cross-sectional preoperative CT scan image at L3 vertebrae is used to outline the area of skeletal muscle or of bilateral psoas muscles. Mean PMA or SM-RA (HU) are calculated to assess muscle fat deposition.	Low PMA or SM-RA
Bone mineral density (13)	BMD is the amount of bone mass per unit volume (volumetric density), or per unit area (areal density).	Bone mineral density (BMD)	The cross-sectional, non-contrast plain preoperative CT images were analyzed at the level of the Th11 vertebra. BMD was measured only on trabecular bone with calculation of the average pixel density (HU) within a circle defined as the mid vertebral core sample	Low BMD

**Table 2:** Characteristics of all included studies.

Reference (author, year)	Country	Study period	Type of study	Indication to surgery	Comparator	Parameter	Pre-operative imaging	N° patients	Male Sex, n (%)	Age, median (range) or mean (SD)	BMI (kg/mq), median (range) or mean (SD)
Peng (2011)	USA	2000-2009	Retrospective	CRLM	Sarcopenia, Sarcopenic obesity	TPA, BMI	CT	259. S/NS: 41/218	155 (60). S: 8 (19.5); NS: 147 (67.4); (p<0.001)	58 (12). S: 59 (1.4); NS: 58 (1.2); (p=0.68)	<30 (74); >30 (26)
Van Vledder (2012)	Netherlands	2001-2009	Retrospective	CRLM	Sarcopenia, central obesity	SMM, intra-abdominal fat	CT (perioperative)	196. S/NS: 38/158	120. S/NS: 11/109 (p<0.001)	64.5 (31–86). S/NS: 65.5 (47–84)/65.0 (31–86); (p=0.229)	S: 23.7 (3.0); NS: 26.7 (3.5); (p<0.001)
Harimoto (2013)	Japan	2004-2009	Retrospective	HCC	Sarcopenia	SMM	CT	186. S/NS: 75/111	S/NS: 50/95; (p=0.004)	S: 67 (11); NS: 66 (10); (p=0.55)	S: 20.5(2.4); NS: 24.0 (2.8); (p<0.001)
Lodewick (2014)	Netherlands, Germany, UK	2005-2012	Retrospective	CRLM	Sarcopenia, obesity, sarcopenic obesity	L3 MI, BMI	CT (within 3 months before surgery)	171. S/NS: 80/91. O/NO: 69/102. SO/NSO: 49/102	NA	64 (24–86). S/NS: 65 (39–86)/64 (24–83); (ns). O/NO: 66 (41–86)/64 (24–83); (ns). SO/NSO: 67 (41–86)/64 (24–83); (ns)	25.7 (18.4-42.8)
Itoh (2014)	Japan	2004-2009	Retrospective	HCC	visceral fat area	VFA	CT	190. Low/High VFA: 84/106	Low/High-VFA: 67 (80)/69 (65); (p=0.395)	Low/High-VFA: 68 (34–87)/69 (31–83); (p=0.918)	Low/High-VFA: 20.5 (14.2–26.1)/24.0 (18.7–32.1); (p<0.001)
Valero III (2014)	USA	2000-2013	Retrospective	Primary liver tumors (HCC, ICC)	Sarcopenia	TPA, TPV	CT (<60 days before surgery or <10 days after)	96. S (TPA)/S (TPV): 44 (45.8)/47 (48.9); (p=0.12)	59 (61.5)	61.9 (12.3)	27.4 (5.4)
Levolger (2015)	Netherlands	2002-2013	Retrospective	HCC	Sarcopenia	SMI	CT (<3 months prior to or >3 days after treatment)	90; resected: 71 (67.8). SR/NSR: 36 (69.2)/25 (65.8)	S/NS: 39 (75.0)/24 (63.2); (p=0.226)	S/NS: 61 (22–86)/62 (25–77); (p=0.48)	S/NS: 24.7 (17.6–33.4)/26.0 (19.5–37.8); (p=0.003)
Otsuji (2015)	Japan	2008-2014	Retrospective	pCCA	Sarcopenia	TPA	preoperative abdominal/pelvic CT <30 days prior to surgery	256. S/NS: 85/171	162 (63). S/NS: 461 (58)/66.1 (107); (p=0.954)	67 (34–85). S/NS: 69 (9)/67 (10); (p=0.133)	21.5 (15.1–30.8). S/NS: 20.5 (2.3)/22.0 (2.7); (p<0.001)
Voron (2015)	France	2006-2012	Retrospective	HCC	Sarcopenia	SMI	CT (<2 months before surgery or <7 days after surgery)	109. S/NS: 59/50	92 (84.4); S/NS: 53 (90)/39 (78); (p=0.09)	61.6 (13.3). S/NS: 64.55 (12.92)/58.25 (13.08); (p=0.013)	25.64 (4.49). S/NS: 24.62 (4.67)/26.85 (3.98); (p=0.009)
Coelen (2015)	Netherlands	1998-2013	Retrospective	pCCA	Total skeletal muscle mass at L3	low skeletal muscle mass	CT	100 (normal/low SMM: 58/42)	normal/low SMM: 36/28; (p=0.636)	normal/low SMM: 62 (9)/61 (11); (p=0.520)	normal/low SMM: 26 (3)/24 (3); (p=0.001)
Hamaguchi (2015)	Japan	2005-2014	Retrospective	HCC	Sarcopenia (skeletal muscle quality)	IMAC	CT	477. High/normal-IMAC: 209/268	389 (82). High/normal-IMAC: 171 (82)/218 (81); (p=0.8944)	67 (10). High/normal-IMAC: 71 (7)/64 (10); (p<0.0001)	23.1 (3.4). High/normal-IMAC: 23.9 (3.6)/22.6 (3.1); (p=0.0002)
Harimoto (2016)	Japan	2004-2013	Retrospective	HCC	Sarcopenia	L3-SMA	CT	296. S/NS: 57/82	S/NS: 40/58; (p=0.943)	S/NS: 76.5 (3.9)/75.9 (4.0); (p= 0.345)	S/NS: 23.3 (3.2)/23.0 (3.5); (p=0.6445)
Kamachi (2016)	Japan	2005-2013	Retrospective	HCC	Sarcopenia	L3-SMI	CT	92. S/NS: 61/31. LR: 35 (57)/11 (35); (p=0.05). RFA 26 (43)/20 (65)	S/NS: 51 (84)/14 (45); (p<0.01)	S/NS: 73 (49–84)/70 (47–80); (p=0.14)	S/NS: 21.2 (14.6–29.7)/24.6 (19.4–33.6); (p<0.01)
Takagi (2016)	Japan	2007 - 2013	Retrospective	HCC	Sarcopenia, ASA score	SMI	CT <3 months prior to surgery	254. S/NS: 118/136	207. S/NS: 93/114; (p=0.31)	65.7 (10.5). S/NS: 68.6 (10.0)/63.1 (10.3); (p<0.001)	23.7 (3.5). S/NS: 22.0 (3.0)/25.2 (3.3); (p<0.001)
Higashi (2016)	Japan	2007 - 2013	Retrospective	Any malignancy requiring liver resection	Sarcopenia, Visceral fat amount	L3-SMI, L3-VFA	CT	144. S/NS: 72/72. Low/High VFA: 75/69	108. S/NS: 54/54 (ns). Low/High VFA: 55/53 (ns)	65.1 (10.1). S/NS: 64.9 (11.6)/65.4 (8.4); (p=0.95). Low/High VFA: 65 (10.4)/65 (9.7); (p=0.99)	22.9 (4.2). S/NS: 21.6 (4.7)/24.2 (3.2); (p=0.0012). Low/High VFA: 21.3 (4.4)/24.7 (3.2); (p<0.001)
Hamaguchi (2016)	Japan	2005 - 2014	Retrospective	HCC	Sarcopenia, quality of skeletal muscle	PMI, IMAC	CT	492. High/normal-IMAC: 219/273	403 (82). High/normal-IMAC: 180 (82)/223 (82); (p=0.885)	68 years (31–89). High/normal-IMAC: 71 (8)/64 (10); (p<0.001)	23.2 (3.4). High/normal-IMAC: 23.9 (3.6)/22.6 (3.1); (p<0.001)
Yabusaki (2016)	Japan	2003-2014	Retrospective	HCC	Sarcopenia	SMI, VFA	CT	195. Low/high-SMI: 89/106	157 (80). Low/high-SMI: 57(64)/100(94); (p<0.001)	66 (22-80). Low/high-SMI: 66.2 (10.1)/63.8 (10.1); (p=0.100)	23.2 (14.3-37.3); Low/high-SMI: 21.8 (2.6)/24.3 (3.5); (p<0.001)
Kobayashi (2016)	Japan	2007-2012	Retrospective	HCC	Quality and quantity of skeletal muscle	IMAC, PMI (ΔIMAC, ΔPMI)	CT	241. Preoperative normal IMAC: 112 (High/Low ΔIMAC 6 M: 32/43. High/Low ΔPMI 6 M: 24/51)	NA	NA	NA
Kobayashi (2017)	Japan	2005-2015	Retrospective	HCC	Sarcopenia, obesity, sarcopenic obesity	SMI, BMI	CT	465. NN/NO/SN/SO: 184 (39.5)/ 219 (47.1)/31 (6.7)/31 (6.7)	367 (78.9). NN/NO/SN/SO: 124 (67.4)/188 (85.8)/24 (77.4)/31 (100); (p<0.001)	67.6 (9.60). NN/NO/SN/SO: 66.0 (10.1)/67.9 (9.2)/69.5 (9.0)/73.6 (7.8); (p<0.001)	23.4 (3.6). NN/NO/SN/SO: 21.7 (2.4)/25.6 (3.3)/18.7 (2.1)/22.5 (2.9); (p<0.001)
Okumura (2017)	Japan	2004-2015	Retrospective	iCCA	Skeletal muscle mass, skeletal muscle quality, visceral adiposity	SMI, MA, VSR	CT (<2 months before operation)	109. Low/High SMI: 69/40. Low/High MA: 53/56. Low/High VSR: 23/86	67 (61). Low/High SMI: 45/22 (p=0.291). Low/High MA: 35/32 (p=0.340). Low/High VSR: 11/56 (p=0.130)	68 (61–73). Low/High SMI: 69 (63–74)/64.5 (55–71); (p=0.027). Low/High MA: 69 (65–77)/64 (54–71); (p<0.001). Low/High VSR: 63 (48-75)/68 (63–73); (p=0.137)	21.9 (19.9–24.4). Low/High SMI: 21.4 (19.6–23.9)/22.7 (20.4–25.8); (p=0.042). Low/High MA: 23.3 (20.9–25.2)/21.1 (19.2–22.4) (p<0.001). Low/High VSR: 21.2(19.2–23.3)/22.1 (20.3–24.4); (p=0.333)
Eriksson (2017)	Sweden	2010-2014	Retrospective	CRLM	Muscle mass	SMI (if NAC muscle mass loss/no loss).	CT	225. S/NS: 147/78. NAC: 97 (43). NAC muscle loss/no loss: 50/47	S/NS: 94 (64)/39 (50); (p=0.043). NAC muscle loss/no loss: 34/27 (ns).	S/NS: 69.0 (63–76)/67.5 (62–72); (p=0.107). NAC muscle loss/no loss: 66.5 (63–75)/68.0 (62–71); (ns)	S/NS: 24.2 (21.5–26.1)/27.6 (25.4–29.9); (p<0.001). NAC muscle loss/no loss: 24.6 (21.4–27.0)/24.3 (22.9–27.1); (ns)
Okuno (2018)	USA	2009-2013	Retrospective	CRLM	sarcopenia, muscle mass, before and after NAC	SMI, muscle loss, body weight	CT (<30 days before preoperative chemotherapy and during the period between preoperative chemotherapy and surgery)	169. Low/High SMI before NAC: 58/111. Low/High SMI after NAC: 61/108	97 (57.4). Minor/major muscle loss: 86 (58.5)/11 (50.0); (p=0.49)	56.2 (11.7)	NA
Chakedis (2018)	USA	2007-2016	Retrospective	BTC	Sarcopenia	PMI	CT (<90 days of the operation)	117. NSR/SR/U: 48/30/39	52 (44). NSR/SR/U: 20 (42)/14 (47)/17 (44); (p=0.910)	66 (50–82). NSR/SR/U: 68 (49–87)/67 (57–77)/64 (47–81); (p=0.929)	28.1 (6.4). NSR/SR/U:29.1 (6.6)/27.7 (6.6)/27.4 (5.9); (p=0.410)
Kroh (2018)	German/Netherlands	2010-2014	Retrospective	HCC	Sarcopenia, obesity, sarcopenic obesity	L3 MI, BFA	CT (<3 months before resection)	70. S/NS: 33/37. O/NO: 28/42. SO/NSO: 21/49	49 (70). S/NS: 21 (64)/28 (76); (p=0.3061). O/NO: 20 (71)/29 (69); (p=1.000). SO/NSO: 15 (71)/34 (69); (p=1.000)	67.74 (12.95). S/NS: 66.97 (16.84)/68.43 (8.23); (p=0.6530). O/NO: 69.18 (12.14)/66.79 (13.53); (p=0.4432). SO/NSO: 69.67 (13.28)/66.92 (12.86); (p=0.4283)	26.64 (4.62). S/NS: 25.29 (4.32)/27.85 (4.61); (p=0.0191). O/NO: 28.61 (4.82)/25.33 (4.04); (p=0.0045). SO/NSO: 26.73 (3.92)/26.61 (4.93); (p=0.9100)
Kobayashi (2018)	Japan	2005-2014	Retrospective	CRLM	Visceral adiposity and muscularity	SMI, IMAC, VSR	CT	124; normal/L-SMI: 100/24. Normal/H-IMAC: 50/74. Normal/H-VSR: 78/46.	78 (63). Normal/L-SMI: 60 (60)/18 (75). Normal/H-IMAC: 33 (66)/45 (61). Normal/H-VSR: 49 (63)/29 (63)	65 (59–70). Normal/L-SMI: 64.5 (58–70)/66.5 (61–75); (p=0.126). Normal/H-IMAC: 60 (53–66)/69 (62–73); (p<0.001). Normal/H-VSR: 63 (55–70)/68 (61–72); (p=0.055)	22.7 (20.3–24.7). Normal/L-SMI: 23.3 (20.5–24.9)/20.9 (18.1–23.5), (p=0.005). Normal/H-IMAC: 21.6 (18.8–23.5)/23.8 (21.0–25.2); (p<0.001). Normal/H-VSR: 22.7 (19.5–24.4)/22.8 (20.4–24.9); (p=0.492)
Miyachi (2019)	Japan	2005-2015	Retrospective	HCC	Bone Mineral Density	BMD at T11	CT (<2months before surgery)	465. Low/normal BMD: 206/161	367	69(62-75). Low/normal BMD: 70 (64–75)/66 (58–74); (p<0.001)	23 (21-25). Low/normal BMD: 23 (21-26)/23 (21-25); (p=0.50)
Berardi (2020)	Italy	2018-2019	Prospective	Any malignancy requiring liver resection	sarcopenia, muscle strength	SMI, handgrip strength measured by a dynamometer	CT (<1 month before surgery)	234. *Group A/B/C/D: 78/13/75/68	158. Group A/B/C/D: 56/10/61/31; (p<0.001)	66.5(58.00-74.25). Group A/B/C/D: 68.00 (59.75-74.00)/66.00 (60.00-73.50)/66.00 (60.00-74.00)/68.00 (54.50-75.00); (p=0.90)	27.12(23.28-29.55). Group A/B/C/D: 27.10 (22.26-30.32)/28.10 (22.7-31.75)/26.34 (25.62-29.70)/25.06 (23.40-31.18); (p=0.11)
Van Dijk (2020)	Canada	NA	Retrospective	CRLM	sarcopenia, myosteatosis, systemic inflammation	SM, SM-RA; VAT; SAT; CRP (z-scores)	CT	97	67 (69). Low/high SM: 39 (65)/28 (76); (p=0.27)	61.2 (10.7). Low/high SM: 63.0 (10.7)/58.3 (10.2); (p=0.035)	NA
Van Dijk (2021)	Netherlands	2008-2013	Retrospective	CRLM	myosteatosis, NAFLD	VAT, SAT, TAT, liver steatosis (0-3). (z-scores)	CT <4 months before surgery	218	142 (65.1)	63.8 (10)	26.3 (3.8)
Okubo (2021)	Japan	2011-2017	Retrospective	HCC	Visceral adiposity	VSR	CT	181. High/low VSR: 60/121	123 (68). High/low VSR: 50 (83)/73 (60); (p=0.001)	67 (34-87). High/low VSR: 68 (53–82)/66 (34–87); (p=0.534)	23.1(13.8-40.4). High/low VSR: 23.2 (17.8-34.2)/22.9 (13.8-40.4); (p=0.573)
Yang (2021)	South Korea	2002-2011	Retrospective	HCC	sarcopenia, myosteatosis, visceral adiposity	PMI, PMA, VATI	CT (<3 months before surgery)	160. S/NS: 28 (17.5)/132 (82.5)	120 (75). S/NS: 17 (60.7)/103 (78.0); (p=0.055)	55 (11). S/NS: 64.07 (8.32)/53.31 (11.22); (p<0.001)	24.26 (2.80). S/NS: 23.57 (2.79)/24.42 (2.81); (p=0.079)
Meister (2022)	Germany	2008-2019	Retrospective	HCC	Sarcopenia, Myosteatosis	SMI, SM-RA	CT (<12 weeks before surgery)	100. MYS/NMYS: 60/40; S/NS: 54/46	72 (72). MYS/NMYS: 42 (70)/30 (75); S/NS: 36 (67)/36 (78); (ns)	67 (11). MYS/NMYS: 70 (8)/64 (14); S/NS: 68 (11)/66 (11); (ns)	26 (22-30). MYS/NMYS 26 (5)/26 (3); (ns). S/NS: 24 (3)/29 (4); (ns)
Martin (2022)	Switzerland	2014-2020	Retrospective	Any liver disease	Sarcopenia	SMI, SMRA	CT (<1 month before surgery)	355. S/NS: 212/143	210 (59.2). S/NS: 139 (65.6)/71 (49.9); (p<0.01)	62 (13.0). S/NS: 64 (13.5)/59 (12.7); (p<0.01)	25.5 (4.9). S/NS: 23.9(3.5)/27.7 (4.9); (p<0.01)

HCC, hepatocellular carcinoma; iCCA, intrahepatic cholangiocarcinoma; pCCA, perihilar cholangiocarcinoma; CRLM, colorectal liver metastases; S, sarcopenia; NS, non-sarcopenia; SMM, skeletal muscle mass; SMI, skeletal muscle index; PMI, psoas muscle index; IMAC, intramuscular adipose content; PMA, psoas muscle attenuation; VATI, visceral adipose tissue index; MA, muscle attenuation; SR-MA, muscle attenuation; VAT, visceral adispose tissue; SAT, subcutaneous adipose tissue; TAT, total adipose tissue; VSR, visceral to subcutaneous adipose tissue area ratio; BTC, biliary tract cancers; NSR, non-sarcopenic resectable; SR, sarcopenic resectable; NA, not assessed.

*Skeletal muscle loss= decrease SMI >5%. *Patients divided in 4 groups: group A (normal muscle mass and strength), group B (reduced muscle strength), group C (reduced muscle mass), group D (reduced muscle mass and strength).

### Does body composition effect short-term outcomes after liver surgery? (Post-operative morbidity, Major-morbidity, liver-specific morbidity, mortality)

#### Liver resection irrespective of indication

Three articles investigated the impact of body composition on the outcome after liver surgery irrespective of the indication for hepatic resection. All authors used sarcopenia as a comparator and specifically focused on the short-term results of liver resection (15–17). (**Supplementary material, Tab.S1**)

Higashi, et al. included in their study 144 patients underwent major hepatectomies (3 or more Couinaud segments) for HCC (63%), CRLM (16%), CCA (13%). Patients were stratified by the presence or absence of sarcopenia and visceral fat amount. No differences were found in overall post-operative morbidity and liver specific morbidity; however, sarcopenic patients showed increased post-operative mortality rates of 9.7% vs. 1.4% in non-sarcopenic patients (p=0.021). In the multivariable analysis, low skeletal muscle mass was confirmed a risk factor for mortality (HR 8.2; p=0.038). In this study, visceral fat amount did not have any impact on surgical outcomes (18).

Berardi, et al. defined sarcopenia as reduced muscle mass and strength as recommended by the European Working Group on sarcopenia in Older People (EWGSOP) and divided a prospective cohort of 244 patients into 4 groups based on the presence of sarcopenia using the SMI and muscle strength (measured by a handgrip strength test) (2). Indications to surgery were represented by HCC (43.2%), CRLM (41.0%), CCA (12.8%), other tumors (3%). The authors reported increased post-operative morbidity in sarcopenic patients while major morbidity and post-operative mortality were not influenced by sarcopenia (16).

By assessing sarcopenia only on CT imaging, Martin et al. did not found difference in post-operative outcomes of 335 patients underwent liver resection. Indications to surgery consisted in CRLM (43.7%), HCC (13.2%), CCA (15.5%); 10% of the patients underwent surgery for non-oncological indications. The authors concluded that other items like muscle strength and physical performance, in addition to imaging, should be considered for preoperative risk stratification (17).

#### Hepatocellular carcinoma

Seventeen articles reported the short-term outcomes (30- or 90-days) of patients underwent hepatectomy for HCC, according to different body composition profiles, their findings are outlined in **Table 3**. The majority (fourteen) used the presence of sarcopenia as comparator (19–35).

**Table 3:** Liver resection for hepatocellular carcinoma (HCC).

Reference (author, year)	Country	Study period	Type of study	Comparator	Parameter	Liver status	N° patients	Major resections (>3 segments), n (%)	Morbidity, n (%)	Liver surgery specific morbidity, n (%)	Liver Failure (ISGLS), n (%)	Major morbidity, n (%)	Mortality, n (%)	DFS/RFS (months), median (range) or (%)	OS (months), median (range) or (%)
Harimoto (2013)	Japan	2004-2009	Retrospective	Sarcopenia	SMM	Cirrhosis: S/NS: 43 (57)/56 (50.5); (ns)	186. S/NS: 75/111	NA	S/NS: 24 (32)/56 (50.5); (p=0.613)	NA	NA	NA	NA	5-y RFS: S/NS: 13/33.2; (p=0.013)	5-y S/NS: 71/83.7; (p=0.001)
Itoh (2014)	Japan	2004-2009	Retrospective	Visceral fat area at umbilicus	VFA	Cirrhosis: Low/High VFA: 33 (39.2)/27 (25.4); (p=0.041)	190. Low/High VFA: 84/106	NA	Low/High VFA: 17 (20.2)/19 (17.9); (p=0.686)	Low/High VFA: 6 (7)/6 (5.6); (p>0.05)	NA	NA	0 (0)	3-/5-/7-y High vs. Low VFA: 54.7/46.4/36.5 vs. 37.3/23.7/14.4; (p=0.001)	3-/5-/7-y High vs. Low VFA: 86.5/78.2/74.3 vs. 79.0/65.3/45.2; (p=0.043)
Levolger (2015)	Netherlands	2002-2013	Retrospective	Sarcopenia	SMI	Cirrhosis: 45 (50.0). S/NS: 26 (50)/19 (50); (p=1.000)	90 (resected: 71 (67.8)). SR/NSR: 36 (69.2)/25 (65.8)	NA	S/NS: 24 (46.2)/13 (34.2); (p=0.255)	NA	NA	S/NS: 17 (32.7)/5 (13.2); (p=0.033)	S/NS: 9 (17.3)/1 (2.6); (p=0.029)	S/NS: 18 (12–24)/18 (11–26); (p=0.67)	S/NS: 33 (17–48)/105 (28–181); (p=0.002)
Voron (2015)	France	2006-2012	Retrospective	Sarcopenia	SMI	Cirrhosis: 63 (57.8). S/NS: 32 (54.2)/31 (62); (p=0.41)	109. S/NS: 59/50	68 (62.4). S/NS: 36 (62)/32 (64); (p=0.75)	41 (37.6). S/NS: 23 (39)/18 (36); (ns)	NA	NA	21 (19.3). S/NS: 13 (22.1)/8 (16); (p=0.560)	5 (4.6). S/NS 4 (6.8)/1 (2)	S/NS: 10.1/34.23; (p<0.001)	S/NS: 52.3/ 70.3; 1-y: 69.8/95.5; (p=0.015)
Hamaguchi (2015)	Japan	2005-2014	Retrospective	Sarcopenia (skeletal muscle quality)	IMAC	Cirrhosis: 477 (100). High/normal IMAC: 209/268; (p=0.0679)	477. High/normal IMAC: 209/268	NA	NA	NA	NA	NA	NA	↓RFS High/normal IMAC; (p=0.0012). Low/normal PMI: ns (p=0.7468)	↓OS High/normal IMAC; (p< 0.0001). Low/normal PMI: ns (p=0.3178)
Harimoto (2016)	Japan	2004-2013	Retrospective	Sarcopenia	L3-SMA	Cirrhosis S/NS: 19/37; (ns)	296. S/NS: 57/82	NA	S/NS: 7 (12.2)/15 (18.3); (p=0.4791)	NA	NA	NA	NA	DFS in patients with sarcopenia also significantly associated with poor prognosis in the older patient group (P=0.030).	↓OS in sarcopenic patients aged ≥70 years (P=0.002).
Kamachi (2016)	Japan	2005-2013	Retrospective	Sarcopenia	SMI	Cirrhosis: 92 (100) (Child A)	92. S/NS: 61/31. Resection: 35 (57)/11 (35); (p=0.05)	NA	NA	NA	NA	NA	NA	1-/3-/5-y RFS S/NS: 39.1/77.1/81.7 vs. 23.5/59.5/75.7; (p=0.03).	1-/3-/5-y S/NS: 91.3/73.6/ 50.5 vs. 96.4/91.6/68.7; (p=0.04)
Takagi (2016)	Japan	2007 - 2013	Retrospective	Sarcopenia, ASA score	SMI	Healthy	254. S/NS: 118/136	97. S/NS: 48/49; (p=0.31)	NA	NA	NA	35. S/NS: 16/19; (p=0.32)	5. S/NS: 1/4; (p=0.13)	NA	S/NS: 58.2/82.4; (p=0.0002). Risk 0 group (NS, ASA<3): 85.6. Risk 1 group (S or ASA>3): 62.4. Risk 2 group (S AND ASA>3): 22.8
Hamaguchi (2016)	Japan	2005 - 2014	Retrospective	Quality and quantity of skeletal muscle	IMAC, PMI	Child A/B: 449 (91)/43 (9)	492. High/normal-IMAC: 219/273	172 (35). High/normal-IMAC: 80 (37)/92 (34); (p=0.513)	180 (37)	82 (16.6)	10 (2.0)	108 (22). High/normal-IMAC: 60 (27)/48 (18); (p=0.009). Low/normal PMI: 36 (36)/72 (20); (p=0.229)	12 (2.4)	NA	NA
Yabusaki (2016)	Japan	2003-2014	Retrospective	Sarcopenia	SMI, VFA	Cirrhosis 195 (100); Child A/B: 182 (93)/13 (7)	195. Low/high-SMI: 89/106	62 (31.8). Low/High-SMI: 25(28)/37(35); (p=0.31)	41 (21.0). Low/High-SMI: 18 (20.2)/23 (21.7); (p=0.80)	NA	NA	NA	5 (2.6). Low/High-SMI: 2 (2.2)/3 (2.8); (p=0.8)	NA	NA
Kobayashi (2016)	Japan	2007-2012	Retrospective	The quality and quantity of skeletal muscle	ΔIMAC, ΔPMI	NA	241. Preoperative normal IMAC: 112 (High/Low ΔIMAC 6 M: 32/43. High/Low ΔPMI 6 M: 24/51)	81 (33.6)	NA	NA	NA	NA	NA	Preoperative normal IMAC: High/Low ΔIMAC 6 M: 19.5/30.3; (p=0.021). High/Low ΔPMI 6 M: 17.9/32.6; (p=0.088). High ΔIMAC (but not High ΔPMI) 6 M (p=0.024) risk factor for HCC recurrence.	NA
Kobayashi (2017)	Japan	2005-2015	Retrospective	Sarcopenia, obesity, sarcopenic obesity	SMI, BMI	Cirrhosis: 239 (51.4). NN/NO/SN/SO: 99 (53.8)/115 (52.5)/15 (48.4)/10 (32.3)	465. NN/NO/SN/SO: 184 (39.5)/219 (47.1)/31 (6.7)/31 (6.7)	175 (37.6). NN/NO/SN/SO: 69 (37.5)/78 (35.6)/12 (38.7)/16 (51.6); (p=0.395)	163 (35.1). NN/NO/SN/SO: 57 (31)/78 (35.6)/15 (48.4)/13 (41.9); (p=0.218)	NA	NA	97 (20.9). NN/NO/SN/SO: 31 (16.9)/44 (20.1)/12 (38.7)/10 (32.3); (p=0.093)	NA	1-/3-y RFS NN/NO/SN/SO: 64.4/37.8 vs. 64.9/38.1 vs. 52.7/31.5 vs.33.8/19.3. ↓SO/NN (p=0.003).	1-, 3-, 5-y: NN/NO/SN/SO 91.1, 78.2, 61.0/91.2, 72.6, 58.2/77.4, 62.7, 38.8/83.9, 45.6, 45.6. ↓SO vs. NN (p=0.002).
Kroh (2018)	German/Netherlands	2010-2014	Retrospective	Sarcopenia, obesity, sarcopenic obesity	L3 MI, BFA	Cirrhosis: 43 (61). S/NS: 18 (55)/25 (68); (p=0.3282). O/NO: 15 (54)/28 (67); (p=0.3211). SO/NSO: 10 (48)/33 (67); (p=0.1800)	70. S/NS: 33/37. O/NO: 28/42. SO/NSO: 21/49	NA	18 (26). S/NS: 8 (24)/10 (27); (p=1.0). O/NO: 7 (25)/11 (26); (p=1.0). SO/NSO: 6 (29)/12 (25); (p=0.7693)	21 (30). S/NS: 7 (21)/14 (38); (p=0.1916). O/NO: 7 (25)/14 (33); (p=0.5961); SO/NSO: 5 (24)/16 (33); (p=0.5745)	4 (6). S/NS: 3 (9)/1 (3); (p=0.3368). O/NO: 2 (7)/2 (5); (p=1.0). SO/NSO: 2 (10)/2 (4) SO; (p=0.5780)	8 (11). S/NS: 4 (12)/4 (11); (p=1.0). O/NO: 3 (11)/5 (12); (p=1.0); SO/NSO: 3 (14)/5 (10); (p=0.6889)	4 (6). S/NS: 1 (3)/3 (8); (p=0.6165). O/NO: 1 (4)/3 (7); (p=0.6450); SO/NSO: 1 (5)/3 (6); (p=1.0)	NA	S/NS/O/NO/SO/NSO: 2.54 (-)/1.44 (1.05-2.07)/2.07 (-)/1.51 (0.93-2.51)/2.63 (-)/1.51 (1.05-2.07). 1-year: 72.7 (59.0-89.6)/67.6 (54.0-84.5)/82.1 (69.1-97.6)/61.9 (48.8-78.5)/81.0 (65.8-99.6)/65.3 (53.3-80.1). 3-y: 45.0 (29.5–68.6)/13.6 (5.6-32.9)/38.5 (22.6-65.5)/20.4 (10.6-39.2)/48.6 (29.0-81.4)/19.5 (10.5-36.0)
Miyachi (2019)	Japan	2005-2015	Retrospective	Bone Mineral Density	BMD at Th11	Low/normal BMD: 145 (70)/127 (79); (p=0.072)	465. Low/normal BMD: 206/161	NA	NA	NA	NA	85(18). Low/normal BMD: 47 (23)/26 (16); (p=0.116)	low BMD associated with a 1.7-fold higher hazard of mortality in males	low BMD not associated with worse RFS in total (p=0.523), male (p=0.809) and female patients (p=0.402).	low BMD associated with worse cancer-specific survival in male patients (p=0.015)
Okubo (2021)	Japan	2011-2017	Retrospective	Visceral adiposity	VSR	Fibrosis(F3) or cirrhosis (F4): 100%	181. High/low VSR: 60/121	11 (6)	34 (19): High/Low VSR: 16(27)/18(15); (p=0.046)	NA	NA	8 (4). High/Low VSR: 5(7)/3(2); (p=0.071)	NA	NA	NA
Yang (2021)	South Korea	2002-2011	Retrospective	Sarcopenia, myosteatosis (IMF), visceral adiposity	PMI, PMA, VATI	Cirrhosis: 86 (53.8). S/NS: 16 (57.1)/70 (53.0); (p=0.692)	160. S/NS: 28 (17.5)/132 (82.5)	NA	NA	NA	NA	NA	72 (45)	3.2 (0.1-15.2). S/NS: ns; (p=0.103). Recurrence S/NS: 16 (57.1)/73 (55.3); (p=0.217)	7.9 (0.1-15.2). S/NS: (p=0.001). Death S/NS: 20 (71.4)/52 (39.4); (p=0.003)
Meister (2022)	Germany	2008-2019	Retrospective	Sarcopenia, Myosteatosis	SMI, SM-RA	Cirrhosis: 42 (42)	100. MYS/NMYS: 60/40; S/NS: 54/46	25 (25)	NA	NA	NA	17 (17). S/NS: 8(15)/9(20). MYS/NMYS: 15(25)/2(5)	6 (6)	DFS S/NS: 35/40; 1-y: 71/89. 3-y: 47/63. 5-y: 27/49; (p=0.118)	MYS/NMYS: 41/60. 1-y: 87/100. 3-y: 57/66. 5-y: 39/48; (p=0.223)

HCC, hepatocellular carcinoma; S, sarcopenia; NS, non-sarcopenia; SMM, skeletal muscle mass; SMI, skeletal muscle index; PMI, psoas muscle index; MA, muscle attenuation; VSR, visceral to subcutaneous adipose tissue area ratio; SR-MA, muscle attenuation; IMAC, intramuscular adipose content; BTC, biliary tract cancers; NSR, non-sarcopenic resectable; SR, sarcopenic resectable; NA, not assessed.

##### Overall morbidity

Nine studies provided data on overall morbidity: in 7 studies, the presence of preoperative sarcopenia (and of sarcopenic obesity in two studies) did not increase morbidity after hepatectomy for HCC (19, 21, 22, 24, 28, 29, 31).

Most of the studies reporting similar post-operative morbidity in sarcopenic and non-sarcopenic patients focused on survival (overall or recurrence-free) as primary endpoint, while the short-term outcomes were listed as secondary endpoints. Sarcopenia was associated with lower BMI and lower serum albumin levels (when reported) in almost all these references, and was more frequent in patients ≥ 60 years old in one study (22). Other baseline characteristics (ASA score, incidence of comorbidities, etiology of underlying liver disease) did not differ substantially according to the presence or absence of sarcopenia. In particular, the prevalence of cirrhosis was 50-100%, equally distributed between sarcopenic and non-sarcopenic patients. Regarding HCC-related factors, there were no significant difference according to the presence/absence of sarcopenia but in few of these studies sarcopenia was associated with more undifferentiated HCC, multinodular HCC, or single HCC >5 cm in size (22, 31). Finally, surgery-related factors, notably the extent of hepatectomy (major hepatectomy), also did not differ substantially in these references.

The relation between visceral adiposity and post-operative complications was investigated by two groups: Itoh, et al. found similar complications in patients with high or low visceral adiposity measured by visceral fat area (VFA) (24). Conversely, in the study by Okubo, et al., visceral adiposity was measured using visceral-to-subcutaneous adipose tissue ratio (VSR). In this study, among 181 cirrhotic patients underwent hepatectomy for HCC, those with high VSR (corresponding to high visceral adiposity) had increased morbidity compared to those with low VSR (p=0.046) (33).

Looking more in detail, only two studies reported the major sources of morbidity (27, 31). According to Hamaguchi et al., pleural effusion, followed by ascites and intra-abdominal abscess, were the most frequent sources of morbidity but the authors did not stratify specific complications according to the presence or absence of sarcopenia. However, at multivariate analysis, preoperative high IMAC (muscle steatosis) was an independent risk factors for increased infectious complications, together with low platelet count, Child-Pugh class B and blood loss (27). Kroh et al. reported that sarcopenic patients developed intra-abdominal abscess (15%) followed by PHLF (9%), bile leak (6%), ascites (6%) and pulmonary complications (6%). In this study specific complication rates were similar compared to non-sarcopenic patients (31). Similarly, in the paper by Itoh et al., HCC patients showing low visceral adiposity, which was correlated with sarcopenia, experienced wound infections (13%), intra-abdominal infections (5.9%) bile leak (3.5%) and ascites (3.5%) but again without significant differences compared to patients presenting high visceral adiposity (20). None of the studies included reported significantly increased infectious complications rate in sarcopenic vs. non-sarcopenic HCC patients.

##### Liver surgery specific morbidity

In two studies neither sarcopenia, sarcopenic obesity nor high visceral adiposity were correlated with liver specific morbidity (20, 31). Interestingly Kroh, et al., determined liver surgery specific morbidity by using a composite endpoint consisting of ascites, post-resection liver failure, bile leakage, intra-abdominal hemorrhage, intra-abdominal abscess (all CD ≥3), operative mortality (36).

##### Major morbidity

Nine studies investigated the relation between body composition profile and major morbidity after hepatectomy for HCC.

Levolger, et al., included 90 patients of which 71 (67.8) underwent surgical resection of HCC. The authors reported increased major morbidity in sarcopenic patients of 32.7% vs. 13.2% in non-sarcopenic patients (p=0.033) (21). Similarly, Hamaguchi, et al. reported increased major morbidity in sarcopenic patients but only when measuring sarcopenia by IMAC (high IMAC) (p=0.009) but not by PMI (low PMI) (p=0.229), suggesting that quality (i.e., muscle steatosis) more than quantity of skeletal muscle impacts the outcomes of liver resection in HCC patients (27).

In the remainders (7 studies), patients with unfavorable body composition profile (sarcopenia, sarcopenic obesity, low bone mineral density, high VSR, myosteatosis) did not show increased major morbidity (22, 26, 29, 31–33, 35). The considerations abovementioned about the inconsistent results on overall morbidity still apply for the outcome major morbidity.

##### Post-operative liver failure

Harimoto, et al. reported an association between sarcopenia and liver dysfunction measured by low albumin and ICG retention rate (19). Specifically concerning post-operative liver failure, in the paper by Kroh, et al., neither sarcopenia nor sarcopenic obesity increased the risk of liver failure in patients underwent hepatectomy for HCC (31).

##### Post-operative mortality

Five studies compared post-operative mortality rates according to the presence or absence of sarcopenia (21, 22, 26, 28, 31). Only Levolger and colleagues showed increased post-operative mortality in sarcopenic vs. non-sarcopenic patients (17.3% vs. 2.6%; p=0.029) (21).

In the context of body composition profiling, sarcopenia is indeed the subject of major interest in the available literature. The presence of pre-operative sarcopenia seems to impair short-term results of hepatectomy for HCC, increasing major morbidity and possibly post-operative mortality but not overall or liver surgery specific morbidity, nor post-hepatectomy liver failure. Some authors also suggested that post-operative outcomes might be influenced by visceral adiposity but data on this subject are still scarce.

#### Cholangiocarcinoma (intra-hepatic, iCCA or peri-hilar, pCCA)

Three studies (two from Japan and one from the Netherlands) specifically analyzed the impact of body composition on the short-term outcomes after liver surgery for iCCA/pCCA (37–39). Two of them focused on the relation between low skeletal muscle mass or sarcopenia and the outcome of major hepatectomy; the authors found a significantly increased risk of liver failure and major morbidity in sarcopenic patients, however in only one study low skeletal muscle mass was associated with increased post-operative mortality. Both these studies did not provide data about overall morbidity nor liver specific morbidity (37, 38). One other study investigated the impact of skeletal muscle mass/quality and visceral adiposity on the outcome of hepatectomy for iCCA without showing significant differences in overall morbidity and mortality for iCCA patients underwent hepatectomy (major hepatectomy 83%). Rates of liver failure, liver specific morbidity and major morbidity were not reported (39).

In this study by Okumura, et al., from Japan, muscle mass quantity and quality were measured respectively by skeletal muscle index (SMI) and muscle attenuation (MA). Subgroup examined consisted in low-vs. high-SMI and low- vs. high-MA patients (low-MA meaning low muscle quality); the four groups showed similar baseline features apart from body composition profile. Of importance, the different cutoff values were established for using receiver operating characteristic (ROC) curves and selected based on the best accuracy in relation to the specific outcome of survival. Failure to show the impact of body composition on post-operative outcomes was not further discussed by the authors (39).

One last study from USA investigated the impact of sarcopenia on 117 patients underwent explorative surgery for biliary tract cancers of which 47 (40.2%) were iCCA or pCCA. With a rate of curative intent hepatic resections of 67% (33% of patients not resected due to metastatic disease at explorative surgery). The authors did not find significant differences in overall morbidity and major morbidity nor in post-operative (30-day) mortality between sarcopenic and non-sarcopenic resectable patients. Despite similar baseline features in the two groups, indications to surgery were heterogeneous (pCCA, iCCA but also gallbladder cancer and distal CCA). Consequently, the surgical procedures performed were also different (ranging from simple cholecystectomy to liver resections, to pancreaticoduodenectomy). These reasons, together with the small size of the population examined, could explain the failure in demonstrating a relation between sarcopenia and post-operative outcome (40). The results are summarized in **Table 4**.

**Table 4:** Liver resection for cholangiocarcinoma (iCCA/pCCA).

Reference (author, year)	Country	Study period	Indication to surgery	Comparator	Parameter	N° patients	Adjuvant treatment, n (%)	Major resections, n (%)	Morbidity, n (%)	Liver specific morbidity, n (%)	Liver Failure (ISGLS), n (%)	Major morbidity, n (%)	Mortality, n (%)	DFS/RFS (months), median (range) or (%)	OS (months), median (range) or (%)
Otsuji (2015)	Japan	2008-2014	pCCA	Sarcopenia	TPA	256. S/NS: 85/171	NA	256 (100)	NA	NA	S/NS: (33)/(16); (p=0.003)	S/NS: 46 (54)/64 (37); (p=0.011)	S/NS: 3 (4)/5 (3); (p=0.793)	NA	NA
Coelen (2015)	Netherlands	1998-2013	pCCA	low skeletal muscle mass	SMM at L3	100. Normal/low SMM: 58/42)	0 (institution policy)	100 (100)	NA	NA	Normal/low SMM: 9 (15.5)/15 (35.7); (p=0.020)	Normal/low SMM: 28 (48.3)/28 (66.7); (p=0.067)	Normal/low SMM: 5 (8.6)/12 (28.6); (p=0.009)	Normal/low SMM: 39.8/43.3; (p=0.748)	Normal/low SMM: 47.5/22.8 (p=0.014). 5-y: 36.2/20.3 (ns)
Okumura (2017)	Japan	2004-2015	iCCA	Skeletal muscle mass, skeletal muscle quality, visceral adiposity	SMI, MA, VSR	109. Low/high SMI: 69/40; Low/high MA: 53/56; Low/high VSR: 23/86	47 (43.1). Low/high SMI: 26 (37.7)/21 (52.5); (p=0.132). Low/high MA: 19 (35.9)/28 (50.0); (p=0.136). Low/high VSR: 15 (65.2)/32 (37.2); (p=0.016)	90 (83). Low/high SMI: 56/34 (p=0.611); Low/high MA: 43/47 (p=0.701); Low/high VSR: 21/69; (p=0.214)	17 (15.6). Low/high SMI: 12 (17.4)/5 (12.5) (p=0.498). Low/high MA: 10 (18.9)/7 (12.5) (p=0.360). Low/high VSR: 1 (4.4)/16 (18.6); (p=0.094)	NA	NA	NA	2 (1.8). Low/high SMI: 2 (2.9)/0 (0.0) (p=0.277). Low/high MA: 1 (1.9)/1 (1.8) (p=0.969). Low/high VSR: 0 (0.0)/2 (2.3); (p=0.460)	↓RFS in low SMI (p=0.015). Low vs. high MA (p=0.233). ↓RFS in high VSR (p=0.049)	↓OS in low SMI (p=0.002). ↓OS in low MA (p=0.032). ↓OS in high VSR (p=0.026)
Chakedis (2018)	USA	2007-2016	BTC	Sarcopenia	PMI	117. NSR/RS: 48/30	NSR/RS: 13 (28)/6 (21); (p=0.792)	NA	NSR/RS: 22 (47)/16 (53); (p=0.164)	NA	NSR/RS: 1 (2)/2 (8); (p=0.251)	NSR/RS: 11 (23)/4 (16); (p=0.50)	NSR/RS: 2 (4)/4 (13); (p=0.139)	NSR/RS time to recurrence: 12.6/7.7 (p=0.504)	NSR/RS: 38.7/12.6; (p=0.006). After recurrence: 55.0/13.5 (p=0.005)

S, sarcopenia; NS, non-sarcopenia; SMM, skeletal muscle mass; SMI, skeletal muscle index; MA, muscle attenuation; VSR, visceral to subcutaneous adipose tissue area ratio; OS, overall survival; RFS, recurrence free survival; DFS, disease free survival; PMI, psoas muscle index; BTC, biliary tract cancers; NSR, non-sarcopenic resectable; SR, sarcopenic resectable; NA, not assessed.

Surgical treatment of CCA mostly rely in major hepatectomy followed by adjuvant chemotherapy (AC) especially in case of pCCA. Available literature suggests increased major morbidity for sarcopenic patients undergoing major hepatectomy for pCCA, however, this seems not to reflect in increased post-operative mortality. On the other side, in case of liver resection for iCCA no clear impact of body composition on short term post-operative outcomes has been showed.

#### Colorectal liver metastases

Five studies investigated the relation of body composition and short term results of hepatectomy in patients with CRLM (41–45). Their findings are summarized in **Table 5**.

**Table 5:** Liver resection for colorectal liver metastases (CRLM).

Reference (author, year)	Country	Study period	Comparator	Parameter	N° patients	Neoadjuvant treatment, n (%)	Adjuvant treatment, n (%)	Major resections (>3 segments), n (%)	Morbidity, n (%)	Liver surgery specific morbidity, n (%)	Liver Failure (ISGLS), n (%)	Major morbidity, n (%)	Mortality, n (%)	DFS/RFS (months), median (range) or (%)	OS (months), median (range) or (%)
Peng (2011)	USA	2000-2009	Sarcopenia, Sarcopenic obesity	TPA	259. S/NS: 41/218	NA	NA	121 (47). S: 19 (46.3); NS: 102 (46.8); (p=0.99)	60 (23)	16 (6)	4 (1.5)	26 (10). S/NS: (22)/(8); (p=0.008). SO/NS: (40)/(8); (p=0.02)	2 (0.8). S: 1 (0.4)	5-y RFS S/NS: 23/27 (p=0.78)	S/NS: (p=0.80). SO/others: 30/46 (p=0.05)
Van Vledder (2012)	Netherlands	2001-2009	Sarcopenia, central obesity	SMM, intra-abdominal fat	196. S/NS: 38/158	91 (86); S: 18 (47); NS: 73 (46.2); (p= 1.000)	0 (institution policy)	63 (32.1)	NA	NA	NA	NA	NA	↓RFS S/NS: 8.7/15.1 (p=0.002). 1-/3-/5-y ↓RFS S vs. NS: 31/20/15 vs. 54.0/36.3/28.5 (p=0.002)	↓OS in S/NS: 23.8/59.8 (p=0.001). 1-/3-/5-y ↓OS in S vs. NS: 84/34/20 vs. 96.2/64.6/49.9 (p< 0.001)
Lodewick (2014)	Netherlands/Germany/UK	2005-2012	Sarcopenia, obesity, sarcopenic obesity	L3 MI, BMI	171. S/NS: 80/91. O/NO: 69/102. SO/NSO: 49/102	NA	NA	S/NS: 33 (41.3)/28 (30.8); (p=0.153). O/NO: 27 (39.1)/34 (33.3); (ns). SO/NSO: 16 (32.7)/45 (36.9); (ns)	S/NS: 33 (41.3)/45 (49.5); (ns). O/NO: 32 (46.4)/46 (45.1); (ns). SO/NSO: 23 (46.9)/55 (45.1); (ns)	S/NS: 16 (20.0)/12 (13.2); (p=0.230); O/NO: 12 (17.4)/16 (15.7); (ns). SO/NSO: 10 (20.4)/18 (14.8); (ns)	NA	S/NS: 21 (26.3)/17 (18.7); (ns). O/NO: 18 (26.1)/20 (19.6); (ns). SO/NSO: 14 (28.6)/24 (19.7); (ns)	1 (0.6)	S/NS: 20 (6–34)/14 (9–18); (ns). O/NO: 22 (2–42)/13 (9–17); (ns). 1-/3-y DFS S vs. NS: 62.7/40.9 vs. 52.3/31.7 (ns). O vs. NO: 65.1/45.5 vs. 51.5/29.5 (ns).	S/NS: 54 (36–71)/49 (34–64); (ns). O/NO: 79 (45–113)/46 (37–57); (p<0.05). 1-/3-/5-y S vs. NS: 92.5/69.0/45.8 vs. 91.2/62.6/40.8 (ns). O vs. NO: 92.8/75.7/60.9 vs. 91.1/59.5/32.4; (p<0.05).
Eriksson (2017)	Sweden	2010-2014	Muscle mass	SMI. If NAC: muscle mass loss/no loss:	225. NAC: 97 (43). S/NS: 147/78. NAC no loss/muscle loss: 50 (51.2)/47 (48.8)	97 (43.1). S/NS: 76 (51.7)/31 (40); (p=0.103)	NAC no loss/muscle loss: 40 (85)/34 (68); (p=0.048)	NA	NA	NA	NA	S/NS: 18 (12.2)/8 (10.2); (p=0.657). NAC no loss/muscle loss: 8/4 (ns)	3 (excluded from survival analysis)	S/NS (ns). A >5% muscle loss during NAC did not result in worse RFS (p=0.105).	↓OS in S (p =0.024). A >5% muscle loss during NAC did not worse OS (p=0.131)
Okuno (2018)	USA	2009-2013	muscle mass and body weight before/after NAC	SMI, body weight	169. Low/High SMI before NAC: 58/111. Low/High SMI after NAC: 61/108	169 (100)	125. minor/major muscle loss: 110 (75.9)/15 (68.2); (p=0.44)	NA	58 (34.3). Minor/major muscle loss: 53 (36.1)/5 (22.7); (p=0.34)	NA	NA	18 (10.6). Minor/major muscle loss: 18 (12.2)/0 (0); (p=0.13)	NA	RFS Low/High SMI before NAC: 10.9/15.3 (p=0.26). RFS Low/High SMI after NAC: 12.5/14.0 (p=0.44). ↓RFS major/minor muscle loss: 9.6/15.9 (p=0.02).	OS Low/High SMI before chemotherapy: 68.4/71.5 (p=0.69). OS Low/High SMI after chemotherapy: 76.6/68.4 (p=0.82). OS major/minor muscle loss: 71.5/68.4 (p=0.42)
Kobayashi (2018)	Japan	2005-2014	Visceral adiposity and muscularity	SMI, IMAC, VSR	124. Normal/L-SMI: 100/24; normal/H-IMAC: 50/74; normal/H-VSR: 78/46.	65 (52). Normal/L-SMI: 54 (54)/11 (46); normal/H-IMAC: 31 (62)/34 (46); normal/H-VSR: 42 (54)/23 (50)	NA	49 (40). normal/L-SMI: 41 (41)/8 (33); normal/H-IMAC: 18 (36)/31 (42); normal/H-VSR: 35 (45)/14 (30)	NA	NA	NA	16 (13. Normal/L-SMI: 13 (13)/3 (12); normal/H-IMAC: 4 (8)/12 (16); normal/H-VSR: 10 (13)/6 (13)	NA	1-/3-/5-y RFS: normal SMI 57.4/29.9/27.6 vs. low SMI 50.0/33.3/25.0 (ns). Normal IMAC 66.0/31.5/28.0 vs. H-IMAC 50.2/30.0/26.3 (ns). Normal VSR 61.1/30.8/28.0 vs. H-VSR 48.4/29.6/24.7 (ns)	1-, 3-, 5-year: Normal SMI 94.7/81.0/64.9 vs. low SMI 95.8/71.2/56.6 (ns). Normal IMAC 91.1/88.5/78.6 vs. H-IMAC 97.3/74.2/50.1 (p=0.054). Normal VSR 96.1/85.2/66.4 vs. and H-VSR 92.7/70.9/54.0 (ns)
Van Dijk (2020)	Canada	NA	sarcopenia, myosteatosis, (systemic inflammation)	SMI, SM-RA; VAT; SAT; (CRP>5mg/dl)	97	46 (47). Low/high SM: 27 (45)/19 (51); (p=0.54)	68 (70). Low/high SM: 45 (75)/23 (62); (p=0.18)	NA	NA	NA	NA	NA	NA	NA	systemic inflammation + one adverse body composition features 43.4 (28.9-57.9) vs. inflammation or one adverse body composition features 79.0 (48.8-109.2) vs. neither inflammation nor one adverse body composition features 109.5 (p=0.010)
Van Dijk (2021)	Netherlands	2008-2013	Myosteatosis	VAT, SAT, TAT.	218	150 (69). NAFLD: 92(68)	NA	NA	NA	NA	NA	NA	NA	NA	NA

S, sarcopenia; NS, non-sarcopenia; SM, skeletal muscle mass; SMI, skeletal muscle index; SR-MA, muscle attenuation; IMAC, intramuscular adipose content; VSR, visceral to subcutaneous adipose tissue area ratio; OS, overall survival; RFS, recurrence free survival; DFS, disease free survival; PMI, psoas muscle index; NSR, non-sarcopenic resectable; SR, sarcopenic resectable, NAFLD, non-alcoholic fatty liver disease, NA, not assessed.

##### Overall morbidity

Three out of these 5 studies reported data on overall morbidity (41, 42, 44).

In the study by Peng, et al. (259 patients, rate of sarcopenia 17%), sarcopenia was associated with overall morbidity at multivariate analysis [odds ratio (OR) 2.22; p=0.02] (41). By contrast, Lodewick et al. in a european population of 171 patients (47% sarcopenic) did not find any significant difference in morbidity for sarcopenic vs. non-sarcopenic patients and sarcopenic obese vs. non-sarcopenic obese patients respectively. The baseline characteristics of these subgroups were similar except for the body composition profile of interest, and the authors underline how different measures and cut-off values for sarcopenia could explain the difference in outcomes within their study and others previously published (42). Okuno, et al. reported similar post-operative complications in a cohort of 169 patients underwent liver resection after NAC, stratified according to the muscle loss during NAC (minor vs. major muscle mass loss). The number of patients able to receive adjuvant chemotherapy were similar in the two groups (110 (75.9) and 15 (68.2); p=0.44). In this study, sarcopenic patients did not have significant muscle loss during NAC (44).

##### Major morbidity

All the 5 studies reported major morbidity rates after LR, with controverse results.

Peng, et al. showed increased major morbidity in sarcopenic and sarcopenic-obese patients vs. non-sarcopenic (22% and 40% vs. 8%; p=0.008 and p=0.02 respectively) (41). On the contrary, Lodewick, et al. found similar incidence of major complications in sarcopenic and sarcopenic-obese patients after LR (42). Similarly, in two other studies involving 225 and 124 patients respectively, major morbidity was similar between sarcopenic and non-sarcopenic patients (43, 45). In the study by Okuno and colleagues muscle mass loss during NAC was not associated with an increase in major complications (p=0.13) (44). Kobayashi and colleagues, apart from sarcopenia measured by SMI, also addressed whether muscle quality, surrogated by intramuscular adipose content (IMAC) and visceral adiposity (visceral-to-subcutaneous adipose tissue ratio (VSR) were associated with major complications with inconsistent results. Of note, high-IMAC (low muscle quality) was significantly associated with older age, higher CA 19-9 blood levels, number of CRLMs>3 and higher BMI; high-VSR (high visceral adiposity) was associated only with number of CRLMs (>3), Conversely, low-SMI was associated only with low BMI but not with CRLM-related factors (size, number) or surgical factors (type of resection, operative time and blood loss), preoperative chemotherapy. Indeed, this study had survival as primary endpoint which also was not impacted neither by low-SMI, nor high-IMAC nor high-VSR. The authors attributed these results to the efficacy of pre-operative chemotherapy (administered to >50% of the population without significant differences according to the cut-off of SMI, IMAC and VSR) and to surgical technique, but they did not further discussed their results on short-term outcomes (45).

##### Liver surgery-specific morbidity

Liver-specific morbidity was not significantly different between sarcopenic vs. non-sarcopenic patients and sarcopenic obese vs. non-sarcopenic obese patients respectively in the study by Lodewick, et al., whose limitations were abovementioned (42).

##### Liver failure and post-operative mortality

The rate of liver failure as well as post-operative mortality after LR for CRLM were not reported in the papers included.

Overall, few studies, mainly focusing on sarcopenia, investigated the impact of body composition on the short-term outcomes of LR for CRLM with controversial results. Specific data are lacking in liver surgery-specific morbidity, post-operative liver failure, post-operative mortality.

### Does body composition impact survival after liver resection?

#### Hepatocellular carcinoma

Fourteen of the articles included analyzed the impact of body composition on survival after liver resection for HCC (**Table 3**). Ten out of 14 used sarcopenia (defined as low quantity or quality of skeletal muscle) as the body composition parameter of reference. Sarcopenia impaired survival in 8 of 10 articles (both OS and RFS/DFS: 5, OS only: 3) (19, 21–26, 34). In 9 studies (OS: 7; RFS/DFS: 5) multivariate analysis confirmed these findings (19, 21, 22, 24–26, 34).

Apart from sarcopenia, multivariate analyses confirmed the predictors of poor survival being those described in existing literature for surgical resection of HCC. Among cancer-related factors: poor differentiation, microvascular invasion, advanced TNM or BCLC stage and multiple HCCs, tumor size and increased alpha-fetoprotein blood levels. Patient-specific factors like sex, age, or ASA score, were less likely related to poor outcome in the studies examined as well as surgery-related factors like intra-operative transfusions or post-operative morbidity. Interestingly, in the populations where pre-operative sarcopenia was associated with poor OS or RFS, the presence of cirrhosis, the extent of surgery (minor vs. major resections) and overall post-operative morbidity were similar between sarcopenic and non-sarcopenic patients.

Assuming that sarcopenia was an independent risk factor for poor survival after hepatectomy for HCC, Kobayashi, et al. further investigated the impact of postoperative changes of quality (IMAC) and quantity of skeletal muscle (PMI) on HCC recurrence. The authors found that in 112 non-sarcopenic patients (preoperative normal IMAC) those with high postoperative impairment of muscle quality had poor RFS (High vs Low ΔIMAC 6 months after hepatectomy: median of 19.5 vs. 30.3 months; p=0.021. High/Low ΔPMI 6 months after hepatectomy: median of 17.9 vs. 32.6 months; p=0.088). In multivariate analysis High ΔIMAC (but not High ΔPMI) 6 months after hepatectomy was a risk factor for HCC recurrence (p=0.024) in non-sarcopenic patients (normal preoperative IMAC) (29). Interestingly, all these studies but two were from eastern countries (Japan or South Korea).

Alongside sarcopenia, the impact of sarcopenic obesity on survival has been investigated in 2 studies with opposite findings. Kobayashi, et al. (465 patients from Japan of whom 31 (6.7%) were sarcopenic obese) found that sarcopenic obese had worse survival compared to non-sarcopenic non-obese patients (median of 39.1 vs. 84.7 months; p=0.002) and worse RFS (median of 8.4 vs. 21.4 months; p=0.003). Multivariate analysis confirmed sarcopenic obesity as a risk factor for both death and HCC recurrence (HR=2.504, p=0.005 and HR=2.031, p=0.006 respectively) (30). In the study by Kroh, et al. (70 patients from Europe of whom 21 (30%) were sarcopenic obese), multivariate analysis could not identify significant difference in postoperative survival regarding sarcopenia or sarcopenic obesity (31).

Visceral adiposity is another parameter of body composition which may impact survival after hepatectomy for HCC. Two studies analyzed the influence of visceral adiposity on survival after hepatectomy for HCC (20, 34). Itoh and colleagues reported worse OS (p=0.001) and DFS (p=0.043) in 84 patients (out of 190) with low visceral adipose content. However, L-VFA was associated with lower BMI, sarcopenia, lower serum albumin, cirrhosis and was not an independent risk factor for survival al multivariate analysis (20). Jang, et al., also reported high visceral adiposity predicting poor OS (HR=1.770; p=0.037) but not RFS (34).

Furthermore, Miyachi and colleagues found low bone mass density (BMD) associated with worse cancer-specific survival only in male patients (p=0.015) underwent hepatectomy for HCC. In males, low BMD was associated with a 1.7-fold higher hazard of mortality (32).

Two studies measured the effect of myosteatosis on survival after liver resection for HCC and both did not find significant correlation (34, 35).

In summary, preoperative sarcopenia impairs survival after hepatectomy for HCC. Other phenotypes such as sarcopenic obesity, high visceral adiposity, low BMD and myosteatosis may impair survival after liver resection of HCC, but few studies are available with controversial results.

#### Cholangiocarcinoma (intra-hepatic or peri-hilar)

Only one study (from the Netherlands) evaluated the impact of low skeletal muscle mass on long-term survival for patients operated of pCCA. With a median follow-up of 28 months (range: 2–122 months) and a median OS of the total cohort (100 patients) of 36.7 months, the authors found that low skeletal muscle mass had a negative impact on OS (p=0.014) but not on DFS (p=0.748) following resection of pCCA. These results were confirmed in the multivariate analysis (38). Okumura, et al. investigated the impact of skeletal muscle mass (SMI) and quality (muscle, attenuation, MA), and visceral adiposity (visceral to subcutaneous adipose tissue area ratio (VSR)) on survival following resection of iCCA. OS was lower in patients with low muscle mass/quality and increased visceral adiposity (low SMI (p=0.002), low MA (p=0.032), high VSR (p=0.026)). Interestingly, a decrease in RFS was observed only low SMI (p=0.015) and in high VSR (p=0.049) patients but not in case of low muscle quality (p=0.233). Multivariate analyses confirmed low SMI (HR: 3.29, p=0.003) and low MA (HR: 2.86, p=0.010) independent risk factors for mortality in patients with stage I–III iCCA, while for stage IV patients only the absence of adjuvant chemotherapy was a risk factor for mortality (39). In the study by Chakedis, et al. (biliary tract cancers of which 40.2% pCCA or iCCA), the authors found sarcopenia being associated with poor OS after resection particularly in patients having disease recurrence. Sarcopenic resectable (SR) patients had shorter survival vs. non-sarcopenic resectable (NSR) patients (12.6 months vs. 38.7 months; p=0.006); moreover, survival in SR patients was comparable to unresectable patients (metastatic disease). The incidence of recurrence (44% in NSR and 53% in SR; p= 0.642) and time-to-recurrence (12.6 in NSR vs. 7.7 months in SR; p =0.504) were not associated with sarcopenia. However, among patients who experienced recurrence, sarcopenic had shorter survival after recurrence (NSR 55.0 months vs. SR 13.5 months; p=0.005) (40).

According to the studies included, an unfavorable body composition profile (i.e., low muscle quality and quantity) may impair the administration of adjuvant chemotherapy or systemic treatment at disease recurrence in these patients, potentially reducing OS. (**Table 4**)

#### Colorectal liver metastases

Six studies specifically addressed whether body composition has an impact on OS and DFS or RFS using the presence of preoperative sarcopenia as comparator (**Table 5**).

Four of these trials found that preoperative sarcopenia had no impact on survival (neither OS nor DFS or RFS) in patients underwent liver resection of CRLM (41, 42, 44, 45).

Of note, in the study by Lodewick the presence of obesity based on body fat percentage seemed to prolong OS (p=0.021) and was identified as an independent predictor (p=0.046) for better OS (the so-called “obesity paradox”) (42).

Poor RFS, but not OS, was observed in patients having major muscle mass loss (≥7%) during NAC versus those having minor muscle mass loss (<7%) in a study from Japan (p=0.002 and p=0.42 respectively) (44).

In addition, Kobayashi and colleagues showed that visceral adiposity and muscle quality had no effect on survival. However, OS but not RFS tended to be lower in patients with high IMAC than in patients with normal IMAC (p=0.054 and p=0.721 respectively) (45).

By contrary, survival was linked to body composition in 3 studies (43, 46, 47). In a retrospective Dutch trial including 196 patients (roughly 20% having sarcopenia), the authors found both reduced RFS and OS of 8.7 vs. 15.1 months (p=0.002) and 23.8 vs. 59.8 months (p=0.001) respectively in sarcopenic vs. non-sarcopenic patients. Preoperative sarcopenia was confirmed an independent predictor of both worse RFS (p=0.002) and OS (p<0.001) (46).

In a study from Sweden, in 147 of 225 patients (65%) preoperative sarcopenia was linked to shorter OS (p =0.024) but not DFS. In the same study, patients with muscle loss >5% during NAC were less likely to undergo adjuvant chemotherapy than others (68% vs 85%, p = 0.048) but a >5% muscle loss during NAC did not worse OS (p=0.131) nor RFS (p=0.105) (43).

Van Dijk et al., showed from a prospective cohort of 289 CRLM patients that NAFLD (p=0.037), myosteatosis (p=0.018), and sarcopenia (p=0.035) were independently associated with shorter OS while high visceral adipose tissue fat content was associated with longer OS (p=0.014). The authors concluded that ectopic fat content in liver as well as skeletal muscle is independently associated with shorter OS in patients undergoing liver resection for CRLM (47). In a previous study including 99 CRLM patients, the same authors advocated a correlation between host-phenotype of adverse body composition features (sarcopenia, low visceral adipose tissue) and systemic inflammation (C-reactive protein >5 mg/mL). This study showed that elevated CRP was more common in patients with sarcopenia (73.8% vs. 51.1%, p=0.029) and the coincidence of elevated CRP and adverse body composition features resulted in worse OS (p=0.008) (48).

The impact of body composition on survival after liver resection of CRLM remains unclear. Few studies are available, and their results are controversial, however interest is growing upon body composition comparators other than skeletal muscle content and sarcopenia.

### Does body composition influence the administration of neoadjuvant or adjuvant treatment in CRLM patients?

Four of the studies included reported the rates of patients receiving NAC. These studies reported similar rates of administration of NAC between sarcopenic and non-sarcopenic patients (43, 45, 46, 48). Also, administration of NAC was similar according to visceral adiposity and muscle quality in the study by Kobayashi, et al. (45) Of importance, none of the studies reported data on the tolerance or completion of NAC according to different body composition profiles.

Two studies specifically have focused on effect of body composition changing during systemic treatment in CRLM patients (43, 44). In the study by Okuno and colleagues muscle mass loss during NAC was not associated with an increase in major complications (p=0.13). However, patients with major muscle mass loss (≥7%) had significantly shorter median RFS than others (9.6 vs. 15.9 months; p=0.02) and on multivariate analysis, major muscle mass loss during NAC was an independent predictor of poor RFS (p=0.045) (44).

In contrast, Erikson et al. reported that major muscle mass loss (>5% compared to the starting value) did not worsened OS (p=0.131) or RFS (p=0.105), although patients with muscle loss >5% during NAC were less likely to undergo adjuvant chemotherapy (AC) than others (68% vs. 85%, p=0.048) impairing the conditions for a complete therapeutic process (43).

In summary, very few studies have evaluated the influence of body composition over the administration of systemic treatment for patients undergoing surgery for CRLM. Available literature reports similar rate of NAC administration between sarcopenic and non-sarcopenic patients, however, there is lack of data about the tolerance and the completion of NAC. Moreover, muscle loss during NAC may shrink the administration of AC but whether this impairs survival or not it remains unclear.

## Discussion

According to the existing evidence, sarcopenia is confirmed to be a pre-operative prognostic factor at least for HCC patients (both short- and long-term outcomes of hepatectomy). These findings are in line with those of a recent meta-analysis on this subject from China on 1420 patients of which 458 presented pre-operative sarcopenia (5). The role of low skeletal muscle mass however remains less clear in the setting of iCCA, pCCA and CRLM. Available data suggest again a potential impact on the outcomes of liver surgery. On the contrary, very few studies investigated the prognostic role of other type of body composition profiles such as the low skeletal muscle quality, sarcopenic obesity, central obesity, or high visceral fat amount, myosteatosis, and low bone mineral density which may also influence the outcomes of hepatectomy for cancers.

This review presents some limitations. First, the studies included are almost only retrospective trials (all but one, a prospective nonrandomized trial).

Second, there is a great heterogeneity of body composition comparator and parameters used in the literature which make difficult to draw conclusions. Sarcopenia indeed is the subject of most of the references. The European Working Group on Sarcopenia in Older People (EWGSOP) recommends using the presence of both low muscle mass and low muscle function (strength or performance) for the diagnosis of sarcopenia. This definition has been recently updated to include physical performance as an indicator of severity (1, 2). While the low quality or quantity of skeletal muscle can be assessed on CT scan imaging by several but similar methods, low muscle function is much more difficult to be measured. It is important to note that despite the recommendations, most of the authors simply refer to CT scan imaging to identify sarcopenic patients, and the measure of muscle function is rarely included in articles. Maybe even more important, there is a heterogeneity in the measurement and cut-off values used in defining the body composition phenotypes which also reflects the heterogeneity of the populations studied (i.e., different cut-off and measurement for sarcopenia in Asian vs. Western populations resulting in different rates of sarcopenia among the studies).

Third, specifically concerning the setting of HCC, most of the studies did not stratify the populations included by the etiology of the underlying liver disease leading to cirrhosis and ultimately to HCC. More in detail, 8 studies provided partial stratification, based on the presence of underlying viral (HBV or HCV) versus others (non-specified) etiologies. Only two studies provided in detail such stratification, reporting hepatitis (HBV and HCV) as the most common etiology followed by metabolic conditions like non-alcoholic fatty liver disease (NAFLD) and non-alcoholic steato-hepatitis (NASH) (22, 35). Unfortunately, none of them investigated any specific correlation between body composition and the presence of NASH. Only Voron et. al, reported NASH being more frequently observed in non-sarcopenic (14%) vs. sarcopenic patients (7%) (22). Therefore, it remains unclear whether the etiology of the underlying liver disease could have an impact on the outcome of surgery in HCC patients showing unfavorable body composition features. Finally, a limitation of the review process used is the absence of any quantitative analysis, due to the large heterogeneity of studies and which we did not performed to be inclusive and comprehensive as much as possible.

### Future research

Future studies should have a prospective design and focus on other body composition profiles different than sarcopenia.

Given the changing epidemiology of primary liver cancer in the last decades, with the rising of metabolic conditions leading to liver disease and cirrhosis all over the world, future studies should also provide stratification of the populations included according to the etiology of the underlying liver disease and investigate its potential relation with the outcomes of liver resection (49–51).

In the specific setting of CRLM, most of the patients are expected to receive neoadjuvant or conversion systemic treatment and/or adjuvant systemic treatment, similarly most of CCA patients are expected to receive adjuvant chemotherapy. It is of mainstay importance to report not only about the rate but also about the tolerance and the compliance to the administration of the systemic treatments which may differ according to different body compositions.

Once identified the patients at high risk of poor outcomes after liver surgery due to their profile of body composition, the next step forward should be to investigate the effectiveness of physical and nutritional pre-habilitation administration to these patients. To our knowledge, one single center randomized clinical trial is currently recruiting patients to elucidate whether preoperative exercise and nutrition improves outcomes of sarcopenic patients undergoing major hepatic surgery for primary or secondary liver cancers. According to the protocol, sarcopenia is diagnosed by both qualitative and quantitative analysis, and patients are randomized in two arms (preoperative nutrition and exercise pre-habilitation followed by major liver resection vs. upfront major liver resection). The primary endpoint is overall 90-morbidity, while survival analysis will be included in the secondary endpoints. The results of this trial are expected to be published in early 2024 (52).

Lastly, whether a minimally invasive approach (MILS) for oncologic liver resection could reduce the impact of an unfavorable body composition profile by reducing for example abdominal wall trauma has not been specifically addressed. The rate of MILS adoption in the studies included is scarcely reported (seven out of 33 articles included) and subgroup outcomes have been reported in only two studies without significant findings (16, 17). This indeed would represent an interesting field of research.

## Conclusions

Body composition profiling is gaining popularity as pre-operative prognostic factor in patients undergoing hepatectomy for cancer; its role has been established at least in the setting of HCC. Sarcopenia is the most exploited body composition parameter. Future research should focus on body phenotypes other than sarcopenia, on the tolerance and compliance to the administration of systemic treatment combined with surgical resection. The potential mitigation of MILS and pre-habilitation programs on the impact of unfavorable body composition, should also be investigated.

## Author contributions

Conception and design of the work: LB, RR, and AC. Acquisition of data: LB, RR, and FV. Data analysis: LB, RR, and FV. Data interpretation: LB, RR, AC, and PM-H. Drafting the article: LB, FV, AC, and PM-H. Critical revision of the article and final approval of the version to be published: all authors. All authors contributed to the article and approved the submitted version.

## References

[1] Cruz-Jentoft AJ Baeyens JP Bauer JM Boirie Y Cederholm T Landi F. Sarcopenia: European consensus on definition and diagnosis: Report of the European working group on sarcopenia in older people. Age Ageing. (2010) 39(4):412–23. doi: 10.1093/ageing/afq034 PMC2886201 20392703

[2] Cruz-Jentoft AJ Bahat G Bauer J Boirie Y Bruyère O Cederholm T. Sarcopenia: Revised European consensus on definition and diagnosis. Age Ageing. (2019) 48(1):16–31. doi: 10.1093/ageing/afy169 30312372 PMC6322506

[3] Zhang G Meng S Li R Ye J Zhao L. Clinical significance of sarcopenia in the treatment of patients with primary hepatic malignancies, a systematic review and meta-analysis. Oncotarget. (2017) 8(60):102474–85. doi: 10.18632/oncotarget.19687 PMC5731973 29254263

[4] Cao Q Xiong Y Zhong Z Ye Q. Computed tomography-assessed sarcopenia indexes predict major complications following surgery for hepatopancreatobiliary malignancy: A meta-analysis. Ann Nutr Metab (2019) 74(1):24–34. doi: 10.1159/000494887 30513518

[5] Xu L Jing Y Zhao C Zhang Q Zhao X Yang J. Preoperative computed tomography-assessed skeletal muscle index is a novel prognostic factor in patients with hepatocellular carcinoma following hepatectomy: A meta-analysis. J Gastrointest Oncol (2020) 11(5):1040–53. doi: 10.21037/jgo-20-122 PMC7657831 33209496

[6] Cornet M Lim C Salloum C Lazzati A Compagnon P Pascal G. Prognostic value of sarcopenia in liver surgery. J Visc Surg (2015) 152(5):297–304. doi: 10.1016/j.jviscsurg.2015.08.001 26476674

[7] Marasco G Serenari M Renzulli M Alemanni LV Rossini B Pettinari I. Clinical impact of sarcopenia assessment in patients with hepatocellular carcinoma undergoing treatments. J Gastroenterol (2020) 55(10):927–43. doi: 10.1007/s00535-020-01711-w PMC7519899 32748172

[8] Perisetti A Goyal H Yendala R Chandan S Tharian B Thandassery R. Sarcopenia in hepatocellular carcinoma: Current knowledge and future directions. World J Gastroenterol (2022) 28(4):432–48. doi: 10.3748/wjg.v28.i4.432 PMC8790553 35125828

[9] Fujiwara N Nakagawa H Kudo Y Tateishi R Taguri M Watadani T. Sarcopenia, intramuscular fat deposition, and visceral adiposity independently predict the outcomes of hepatocellular carcinoma. J Hepatol (2015) 63(1):131–40. doi: 10.1016/j.jhep.2015.02.031 25724366

[10] Ha Y Kim D Han S Chon YE Lee YB Kim MN. Sarcopenia predicts prognosis in patients with newly diagnosed hepatocellular carcinoma, independent of tumor stage and liver function. Cancer Res Treat (2018) 50(3):843–51. doi: 10.4143/crt.2017.232 PMC6056958 28882021

[11] Ross R Neeland IJ Yamashita S Shai I Seidell J Magni P. Waist circumference as a vital sign in clinical practice: a consensus statement from the IAS and ICCR working group on visceral obesity. Nat Rev Endocrinol (2020) 16(3):177–89. doi: 10.1038/s41574-019-0310-7 PMC7027970 32020062

[12] Miljkovic I Vella CA Allison M. Computed tomography-derived myosteatosis and metabolic disorders. Diabetes Metab J (2021) 45(4):482–91. doi: 10.4093/dmj.2020.0277 PMC8369205 34352985

[13] Pickhardt PJ Pooler BD Lauder T del Rio AM Bruce RJ Binkley N. Opportunistic screening for osteoporosis using abdominal computed tomography scans obtained for other indications. Ann Intern Med (2013) 158(8):588. doi: 10.7326/0003-4819-158-8-201304160-00003 23588747 PMC3736840

[14] Rahbari NN Garden OJ Padbury R Brooke-Smith M Crawford M Adam R. Posthepatectomy liver failure: A definition and grading by the international study group of liver surgery (ISGLS). Surgery. (2011) 149(5):713–24. doi: 10.1016/j.surg.2010.10.001 21236455

[15] Higashi T Hayashi H Taki K Sakamoto K Kuroki H Nitta H. Erratum to: Sarcopenia, but not visceral fat amount, is a risk factor of postoperative complications after major hepatectomy. Int J Clin Oncol (2017) 22(5):986–90. doi: 10.1007/s10147-017-1163-5 28755069

[16] Berardi G Antonelli G Colasanti M Meniconi R Guglielmo N Laurenzi A. Association of sarcopenia and body composition with short-term outcomes after liver resection for malignant tumors. JAMA Surg (2020) 155(11):e203336. doi: 10.1001/jamasurg.2020.3336 32965483 PMC7512123

[17] Martin D Maeder Y Kobayashi K Schneider M Koerfer J Melloul E. Association between CT-based preoperative sarcopenia and outcomes in patients that underwent liver resections. Cancers. (2022) 14(1):261. doi: 10.3390/cancers14010261 35008425 PMC8750804

[18] Higashi T Hayashi H Taki K Sakamoto K Kuroki H Nitta H. Sarcopenia, but not visceral fat amount, is a risk factor of postoperative complications after major hepatectomy. Int J Clin Oncol (2016) 21(2):310–9. doi: 10.1007/s10147-015-0898-0 26338271

[19] Harimoto N Shirabe K Yamashita YI Ikegami T Yoshizumi T Soejima Y. Sarcopenia as a predictor of prognosis in patients following hepatectomy for hepatocellular carcinoma. Br J Surg (2013) 100(11):1523–30. doi: 10.1002/bjs.9258 24037576

[20] Itoh S Shirabe K Matsumoto Y Yoshiya S Muto J Harimoto N. Effect of body composition on outcomes after hepatic resection for hepatocellular carcinoma. Ann Surg Oncol (2014) 21(9):3063–8. doi: 10.1245/s10434-014-3686-6 24719020

[21] Levolger S van Vledder MG Muslem R Koek M Niessen WJ de Man RA. Sarcopenia impairs survival in patients with potentially curable hepatocellular carcinoma: Sarcopenia impairs survival in HCC. J Surg Oncol (2015) 112(2):208–13. doi: 10.1002/jso.23976 26266324

[22] Voron T Tselikas L Pietrasz D Pigneur F Laurent A Compagnon P. Sarcopenia impacts on short- and long-term results of hepatectomy for hepatocellular carcinoma. Ann Surg (2015) 261(6):1173–83. doi: 10.1097/SLA.0000000000000743 24950264

[23] Hamaguchi Y Kaido T Okumura S Ito T Fujimoto Y Ogawa K. Preoperative intramuscular adipose tissue content is a novel prognostic predictor after hepatectomy for hepatocellular carcinoma. J Hepato-Biliary-Pancreat Sci (2015) 22(6):475–85. doi: 10.1002/jhbp.236 25755128

[24] Harimoto N Yoshizumi T Shimokawa M Sakata K Kimura K Itoh S. Sarcopenia is a poor prognostic factor following hepatic resection in patients aged 70 years and older with hepatocellular carcinoma: Sarcopenia in elderly patients with HCC. Hepatol Res (2016) 46(12):1247–55. doi: 10.1111/hepr.12674 26880049

[25] Kamachi S Mizuta T Otsuka T Nakashita S Ide Y Miyoshi A. Sarcopenia is a risk factor for the recurrence of hepatocellular carcinoma after curative treatment. Hepatol Res (2016) 46(2):201–8. doi: 10.1111/hepr.12562 26223826

[26] Takagi K Yagi T Yoshida R Shinoura S Umeda Y Nobuoka D. Sarcopenia and American society of anesthesiologists physical status in the assessment of outcomes of hepatocellular carcinoma patients undergoing hepatectomy. Acta Med Okayama. (2016) 70(5):8. doi: 10.18926/AMO/54594 27777428

[27] Hamaguchi Y Kaido T Okumura S Kobayashi A Fujimoto Y Ogawa K. Muscle steatosis is an independent predictor of postoperative complications in patients with hepatocellular carcinoma. World J Surg (2016) 40(8):1959–68. doi: 10.1007/s00268-016-3504-3 27071610

[28] Yabusaki N Fujii T Yamada S Suzuki K Sugimoto H Kanda M. Adverse impact of low skeletal muscle index on the prognosis of hepatocellular carcinoma after hepatic resection. Int J Surg (2016) 30:136–42. doi: 10.1016/j.ijsu.2016.04.049 27154615

[29] Kobayashi A Kaido T Hamaguchi Y Okumura S Taura K Hatano E. Impact of postoperative changes in sarcopenic factors on outcomes after hepatectomy for hepatocellular carcinoma. J Hepato-Biliary-Pancreat Sci (2016) 23(1):57–64. doi: 10.1002/jhbp.302 26572789

[30] Kobayashi A Kaido T Hamaguchi Y Okumura S Shirai H Yao S. Impact of sarcopenic obesity on outcomes in patients undergoing hepatectomy for hepatocellular carcinoma. Ann Surg (2019) 269(5):924–31. doi: 10.1097/SLA.0000000000002555 29064889

[31] Kroh A Uschner D Lodewick T Eickhoff RM Schöning W Ulmer FT. Impact of body composition on survival and morbidity after liver resection in hepatocellular carcinoma patients. Hepatobiliary Pancreat Dis Int (2019) 18(1):28–37. doi: 10.1016/j.hbpd.2018.07.008 30115516

[32] Miyachi Y Kaido T Yao S Shirai H Kobayashi A Hamaguchi Y. Bone mineral density as a risk factor for patients undergoing surgery for hepatocellular carcinoma. World J Surg (2019) 43(3):920–8. doi: 10.1007/s00268-018-4861-x 30465085

[33] Okubo S Shindoh J Kobayashi Y Umino R Akabane M Kojima K. Adipose tissue distribution predicts prognosis of cirrhotic patients undergoing hepatectomy for hepatocellular carcinoma. Ann Surg Oncol (2021) 28(11):6738–46. doi: 10.1245/s10434-021-09658-9 33554286

[34] Jang HY Choi GH Hwang SH Jang ES Kim JW Ahn JM. Sarcopenia and visceral adiposity predict poor overall survival in hepatocellular carcinoma patients after curative hepatic resection. Transl Cancer Res (2021) 10(2):854–66. doi: 10.21037/tcr-20-2974 PMC8799077 35116415

[35] Meister FA Lurje G Verhoeven S Wiltberger G Heij L Liu WJ. The role of sarcopenia and myosteatosis in short- and long-term outcomes following curative-intent surgery for hepatocellular carcinoma in a European cohort. Cancers. (2022) 14(3):720. doi: 10.3390/cancers14030720 35158988 PMC8833751

[36] van den Broek MAJ van Dam RM van Breukelen GJP Bemelmans MH Oussoultzoglou E Pessaux P. Development of a composite endpoint for randomized controlled trials in liver surgery. Br J Surg (2011) 98(8):1138–45. doi: 10.1002/bjs.7503 21557208

[37] Otsuji H Yokoyama Y Ebata T Igami T Sugawara G Mizuno T. Preoperative sarcopenia negatively impacts postoperative outcomes following major hepatectomy with extrahepatic bile duct resection. World J Surg (2015) 39(6):1494–500. doi: 10.1007/s00268-015-2988-6 25651963

[38] Coelen RJS Wiggers JK Nio CY Besselink MG Busch ORC Gouma DJ. Preoperative computed tomography assessment of skeletal muscle mass is valuable in predicting outcomes following hepatectomy for perihilar cholangiocarcinoma. HPB. (2015) 17(6):520–8. doi: 10.1111/hpb.12394 PMC4430783 25726722

[39] Okumura S Kaido T Hamaguchi Y Kobayashi A Shirai H Fujimoto Y. Impact of skeletal muscle mass, muscle quality, and visceral adiposity on outcomes following resection of intrahepatic cholangiocarcinoma. Ann Surg Oncol (2017) 24(4):1037–45. doi: 10.1245/s10434-016-5668-3 27837297

[40] Chakedis J Spolverato G Beal EW Woelfel I Bagante F Merath K. Pre-operative sarcopenia identifies patients at risk for poor survival after resection of biliary tract cancers. J Gastrointest Surg (2018) 22(10):1697–708. doi: 10.1007/s11605-018-3802-1 29855867

[41] Peng PD van Vledder MG Tsai S de Jong MC Makary M Ng J. Sarcopenia negatively impacts short-term outcomes in patients undergoing hepatic resection for colorectal liver metastasis. HPB. (2011) 13(7):439–46. doi: 10.1111/j.1477-2574.2011.00301.x PMC3133709 21689226

[42] Lodewick TM van Nijnatten TJA van Dam RM van Mierlo K Dello SAWG Neumann UP. Are sarcopenia, obesity and sarcopenic obesity predictive of outcome in patients with colorectal liver metastases? HPB (2015) 17(5):438–46. doi: 10.1111/hpb.12373 PMC4402055 25512239

[43] Eriksson S Nilsson JH Strandberg Holka P Eberhard J Keussen I Sturesson C. The impact of neoadjuvant chemotherapy on skeletal muscle depletion and preoperative sarcopenia in patients with resectable colorectal liver metastases. HPB. (2017) 19(4):331–7. doi: 10.1016/j.hpb.2016.11.009 28089364

[44] Okuno M Goumard C Kopetz S Vega EA Joechle K Mizuno T. Loss of muscle mass during preoperative chemotherapy as a prognosticator for poor survival in patients with colorectal liver metastases. Surgery. (2019) 165(2):329–36. doi: 10.1016/j.surg.2018.07.031 30197278

[45] Kobayashi A Kaido T Hamaguchi Y Okumura S Shirai H Kamo N. Impact of visceral adiposity as well as sarcopenic factors on outcomes in patients undergoing liver resection for colorectal liver metastases. World J Surg (2018) 42(4):1180–91. doi: 10.1007/s00268-017-4255-5 28936708

[46] van Vledder MG Levolger S Ayez N Verhoef C Tran TCK IJzermans JNM. Body composition and outcome in patients undergoing resection of colorectal liver metastases19. Br J Surg (2012) 99(4):550–7. doi: 10.1002/bjs.7823 22246799

[47] Dijk DPJ Zhao J Kemter K Baracos VE Dejong CHC Rensen SS. Ectopic fat in liver and skeletal muscle is associated with shorter overall survival in patients with colorectal liver metastases. J Cachexia Sarcopenia Muscle. (2021) 12(4):983–92. doi: 10.1002/jcsm.12723 PMC8350209 34061469

[48] Dijk DPJ Krill M Farshidfar F Li T Rensen SS Olde Damink SWM. Host phenotype is associated with reduced survival independent of tumour biology in patients with colorectal liver metastases. J Cachexia Sarcopenia Muscle. (2019) 10(1):123–30. doi: 10.1002/jcsm.12358 PMC6438330 30378742

[49] Xing QQ Li JM Dong X Zeng DY Chen ZJ Lin XY. Socioeconomics and attributable etiology of primary liver cancer, 1990-2019. World J Gastroenterol (2022) 28(21):2361–82. doi: 10.3748/wjg.v28.i21.2361 PMC9185214 35800181

[50] Japan Etiology of Liver Cirrhosis Study Group in the 54th Annual Meeting of JSH Enomoto H Ueno Y Hiasa Y Nishikawa H Hige S. The transition in the etiologies of hepatocellular carcinoma-complicated liver cirrhosis in a nationwide survey of Japan. J Gastroenterol (2021) 56(2):158–67. doi: 10.1007/s00535-020-01748-x PMC7862502 33219410

[51] Huang DQ Singal AG Kono Y Tan DJH El-Serag HB Loomba R. Changing global epidemiology of liver cancer from 2010 to 2019: NASH is the fastest growing cause of liver cancer. Cell Metab (2022) 34(7):969–977.e2. doi: 10.1016/j.cmet.2022.05.003 35793659 PMC9762323

[52] Berardi G. Nutrition and exercise prehabilitation to reduce morbidity following major liver surgery in sarcopenic patients (NEXPREM). ClinicalTrials (2022) 6.

