# Supplementary material for: Imaging based body composition profiling and outcomes after oncologic liver surgery
Source: Front Oncol. 2022 Dec 8;12:1007771. doi: 10.3389/fonc.2022.1007771 (PMC9773835; doi:10.3389/fonc.2022.1007771)
Supplement: Supplementary file 1 [file Table_1.docx]

**Supplementary material (Table):**

Tab.S1 Liver resection irrespective of indications

| **Reference (author, year)** | **Country** | **Study period** | **Type of study** | **Indication to surgery** | **Comparator** | **Parameter** | **N° patients** | **Major resections (>3 segments), n (%)** | **Morbidity, n (%)** | **Liver surgery specific morbidity, n (%)** | **Liver Failure (ISGLS), n (%)** | **Major morbidity, n (%)** | **Mortality, n (%)** | **DFS/RFS (months), median (range) or (%)** | **OS (months), median (range) or (%)** |
| --- | --- | --- | --- | --- | --- | --- | --- | --- | --- | --- | --- | --- | --- | --- | --- |
| **Higashi (2016)** | Japan | 2007 - 2013 | Retrospective | Any malignancy requiring major LR | Sarcopenia, Visceral fat amount | L3 SMI, L3 VFA | 144. S/NS: 72/72. Low/High VFA: 75/69 | 144 (100) | 43 (30). S/NS: 23 (32)/20 (28); (p=0.58). Low/High VFA: 25 (33)/18 (26); (p=0.5) | 11 (7.6). S/NS: 8 (11)/3 (4.2); (p=0.11). Low/High VFA: 6 (8)/5 (7.2); (p=0.9) | NA | NA | 8 (5.6). S/NS: 7 (9.7)/1 (1.4); (p=0.021). Low/High VFA: 3 (4)/5 (7.2) (p=0.37) | NA | NA |
| **Berardi (2020)** | Italy | 2018-2019 | Prospective | Any malignancy requiring LR | sarcopenia, muscle strength | SMI, handgrip strength (measured by dynamometer) | 234. *Group A/B/C/D: 78/13/75/68 | 64(27.4). Group A/B/C/D: 19 (24.4)/4 (30.8)/19 (25.3)/22 (32.4); (p=0.69) | 72 (30.8). Group A/B/C/D: 5 (6.4)/3 (23.1)/29 (38.7)/35 (51.5); (p<0.001) | NA | NA | 18 (7.7). Group A/B/C/D: 1 (1.3)/0/5 (6.7)/12 (17.6); (p=0.30) | 5 (2.1). Group A/B/C/D: 1 (1.3)/1 (7.7)/0/3 (4.4); (p=0.13) | NA | NA |
| **Martin (2022)** | Switzerland | 2014-2020 | Retrospective | Any liver disease requiring LR | Sarcopenia | SMI, SMRA | 355. S/NS: 212/143 | 190 (53.5). S/NS: 117 (55.2)/73 (51.0); (p=0.450) | S/NS: 98 (46.2)/69 (48.3); (p=0.745) | bile leak: 45 (12.7). S/NS: 20/25 (p=0.156) | 7 (2.1). S/NS: 6/1 (p=0.203) | S/NS: 24 (11.3)/16 (11.3); (p=1.000) | 0 (0) | NA | malignancy: 274 (77.2). S/NS: 15/16 (p=0.867) |

S= sarcopenia; NS= non-sarcopenia; VFA= visceral fat amount; SMI= skeletal muscle index; SMRA= skeletal muscle radiation attenuation. *Patients divided in 4 groups: group A (normal muscle mass and strength), group B (reduced muscle strength), group C (reduced muscle mass), group D (reduced muscle mass and strength).
